# Cancer health literacy in Kenya - A scoping review on evidence, concept and a situational analysis of interventions

**DOI:** 10.3389/fpubh.2025.1527400

**Published:** 2025-05-16

**Authors:** Stefanie Harsch, Lea S. Weber, Dinah Kassaman, Peter N. Kailemia, Victor O. Oria

**Affiliations:** ^1^Center for Medicine and Society, University of Freiburg, Freiburg, Germany; ^2^School of Nursing and Midwifery, Aga Khan University, Nairobi, Kenya; ^3^School of Nursing, Meru University of Science and Technology, Meru, Kenya; ^4^Research Division, Integrated Cancer Research Foundation (ICRF Kenya), Nairobi, Kenya

**Keywords:** health literacy, cancer, cancer health literacy, Kenya, interventions, scoping review

## Abstract

**Background:**

In light of the rising incidences of cancer in Kenya, there is an urgent need to evaluate and strengthen cancer health literacy (CHL). Nevertheless, no review has been undertaken to synthesise the empirical evidence on CHL in Kenya. This study aims to review the evidence, explore the use of the concept CHL and, assess the situation of HL interventions.

**Method:**

A comprehensive scoping review was conducted to explore the evidence on CHL in Kenya. The review included a thorough concept analysis, based on the work of Sorensen et al. and a situational analysis, employing the precede–proceed model of Green and Kreuter. Nine international scientific databases (PubMed, Web of Science, CINAHL, APA PsycINFO, OpenDissertation, ERIC, Cochrane Library, African Journals Online, and African Index Medicus) were searched to identify articles on cancer health literacy-related concepts in Kenya since 2010. Two researchers screened the titles and abstracts and analysed the full texts based on the eligibility criteria. Data was extracted using a deductively developed coding scheme.

**Results:**

A total of 727 articles were identified, of which 110 deemed eligible for analysis. The studies presented findings on the geographical distribution, cancer type, phase of the cancer care continuum, target group, cancer-related aspects, and intervention's influencing factors. Most studies concentrated on early detection and assessed barriers and facilitators. A mere 31 studies reported on treatment. There is a paucity of knowledge regarding educational interventions for cancer patients and their effectiveness. The concept of CHL was primarily concerned with possessing knowledge and information, with relatively little attention devoted to the processes of using them, specifically their appraisal and application. Various situational aspects of interventions were identified.

**Conclusion:**

Further research is required to develop and implement promising interventions for the general public and cancer patients, enabling them to utilise information more effectively. Interventions that are decentralised, digital, and involve cancer patients and survivors are recommended to meet the needs of the growing numbers of cancer patients and their families. The findings can inform the development of promising CHL interventions and mitigate their barriers in Kenya and beyond.

## 1 Introduction

The incidence of cancer is increasing globally, including in sub-Saharan African countries such as Kenya (1, 2). In 2022, Kenya had a total of 44,726 cancer cases with a 5-year prevalence of 102,152 and 29,317 deaths, according to the World Health Organization's International Agency for Research on Cancer (IARC) (2). This makes Kenya the second most affected country in Eastern Africa, after Uganda, and the seventh most affected country in Sub-Saharan Africa (3). Nevertheless, the actual number of cases is likely to be considerably higher than the figures by IARC, due to low screening rates, undetected cases, and under-reporting. In order to effectively address the increasing incidence of cancer, three key challenges must be addressed. Firstly, it is imperative that the healthcare sector expands the provision of cancer services and that more oncology care providers are trained (4). Secondly, the financial burden on healthcare systems is notably high, due to the necessity for additional cancer treatment facilities, oncology specialists, and nurses, as well as the high cost of treatment for cancer patients and their families (5). The Kenyan government has announced plans to expand the range of treatments covered by the National Health Insurance Funds in the new Social Health Insurance Fund from fall 2024 onwards (6). However, a third challenge persists: there is a growing demand for information about cancer. In order to address this issue, the Kenyan Ministry of Health and local cancer organisations, such as the members of the Kenyan Network of Cancer Organisations (KENCO) (7), provide information about cancer in person, online, and through mass media such as radio and television. However, the mere possession of knowledge, awareness, and information is insufficient due to the pervasiveness of misperceptions, information deficiencies and inaccuracies (8–10). Research has repeatedly demonstrated that individuals may lack the requisite competence to understand and apply this knowledge (11). This vital competence is referred to as cancer health literacy (CHL) or cancer literacy when linked to cancer-related health literacy (12). The concept of health literacy is in a constant state of evolution and encompasses the ability to read health information up to a range of abilities, including knowledge, motivation, and all competencies relevant to finding, understanding, appraising, and using information, resources, supports, and environments (13, 14). The latter concept is more multidimensional in nature and is widely applied in the twenty-first century (11). Empirical evidence has demonstrated that individuals with low health literacy are more prone to underutilise health services, to miss out on health promotion and prevention services, to delay help-seeking, to have difficulty communicating with health professionals, to be non-adherent to treatment regimes, and to experience poorer health outcomes (11). It is therefore evident that enhancing health literacy represents a pivotal strategy for addressing public and global health concerns. As such, health literacy is content- and context-specific, e.g., specific to cancer and the Kenyan context. A number of cancer-specific tasks and essential skills have been identified, such as making decisions, evaluating treatment-related information, living with cancer, and dispelling disease-related myths and misconceptions (15, 16). Adequate engagement and performance of these tasks require a high level of cancer health literacy (17). The implementation of these specific tasks also depends on the structures and support systems available in the country in question, the healthcare system in place, and the social context. The term “cancer literacy” is becoming increasingly prevalent in the global discourse (12). To avoid any confusion between cancer literacy and the ability to read and write about cancer, this study will employ the term “cancer health literacy” (CHL). The findings of studies (17–22) on cancer health literacy indicate that individuals with higher levels of CHL are better equipped to cope with the challenges of cancer. They engage in cancer prevention behaviours, experience less depression and anxiety, have lower risk factors, incur lower treatment costs, and ultimately enjoy a better quality of life. Furthermore, enhanced health literacy has been associated with superior treatment outcomes and diminished premature mortality rates.

Unfortunately, health and CHL are low worldwide, including in Kenya (22). This has the effect of impeding cancer control and treatment. Therefore, efforts must be intensified to enhance CHL in an efficacious and sustainable manner. Health literacy is acquired informally in everyday life and can be formally improved through health education and organisational support (23, 24). Numerous studies have been conducted in Kenya on cancer education and related concepts, including cancer health literacy, knowledge, and awareness (25, 26). Each of these studies focused on a specific phase of the cancer care pathway. For example, Huschke et al.'s focused on HPV vaccinations (27), while Baratedi et al. (28) and Mbugua et al. (29) examined breast and prostate cancer screening, respectively. Makau-Barasa et al. (30) concentrated on treatment or palliative phases, whereas Kassaman et al. (22) and Kailemia et al. (31) investigated specific aspects such as psychological factors, barriers, and facilitators at various socio-ecological levels. Despite the growing body of empirical evidence on cancer in Kenya, no review has yet been conducted to explore and summarise the empirical evidence on cancer health literacy, its influencing factors, cancer-related behaviour, and health. This represents a significant shortcoming as a considerable proportion of the research is overlooked, unnecessarily repeated, and thus resources are wasted. To allocate resources in a more targeted manner towards effective health education interventions, it is necessary to summarise the empirical evidence base on cancer health literacy in Kenya. This process should involve learning from existing research, identifying gaps in the current evidence, informing the development of context-specific targeted concepts and interventions, and increasing the studies' visibility globally. In 2017, a scoping review on oncology research in Kenya was conducted (32) to inform the development of the cancer care and control strategy. It included a comprehensive mapping of Kenyan-based research, although the focus was not on cancer health literacy specifically. The Kenyan Ministry of Health continues to encourage the conduct of scoping reviews to obtain a good overview and insight into relevant phenomena (4). The purpose of this study is to present an overview of the **landscape of empirical evidence** on **cancer health literacy** in **Kenya**. To this end, three objectives were identified with the aim of achieving a comprehensive understanding of the concept and the context:

1. To identify and characterise empirical evidence on cancer health literacy of the public and cancer patients in Kenya,
2. to explore what constitutes cancer health literacy of the public and cancer patients in Kenya during the different phases of cancer care and to develop a cancer health literacy model,
3. to describe the situation related to cancer health literacy interventions for the public and cancer patients in Kenya.

This scoping review is part of the research project, “Improving Cancer Health Literacy through Online Storytelling in Sub-Saharan Africa” (CaLioS) (33), which aims to gain insight into CHL and its context in Kenya, explore the potential of storytelling approaches to enhance CHL, develop a website featuring cancer patients' narratives, and evaluate the website's efficacy. The project is financially supported by the German Ministry of Education and Research, through a postdoctoral research project selected by the German Alliance for Global Health Research. The protocol of the scoping review was preregistered on OSF (https://doi.org/10.17605/OSF.IO/JKVE5).

It is crucial to acknowledge that the concept of CHL and its contextual complexities can be described and explored in a multitude of ways. To ensure a comprehensive and systematic approach, two frameworks have been employed for the purpose of identifying, mapping, and analysing the concept within its contextual framework.

### 1.1 Conceptualisation of cancer health literacy

There are diverse conceptualisations of CHL (17, 34, 35). While some researchers focus solely on knowledge related to cancer [e.g., using the instrument CHLT-30 (34)], qualitative studies (22) have demonstrated that it is a more expansive concept, encompassing a broad range of skills and motivation necessary to access, understand, evaluate, and apply cancer-related information (12). This study employed the definition and comprehensive health literacy framework created by the European Consortium on Health Literacy in 2012 (14), which was based on a systematic analysis of health literacy concepts and frameworks. The framework distinguishes between three domains of health literacy, including health promotion, disease prevention, and healthcare, as well as four dimensions of information engagement, namely, accessing, understanding, appraising, and applying health information. This framework was adopted by the World Health Organization (36) and is a valuable tool for guiding discussions on CHL, as it provides a clear differentiation of domains and dimensions (12). This general framework must be operationalised based on the content and context. The content refers to the specific tasks and competencies required to engage with a health concern, in this case cancer. The context refers to the societal and healthcare context in which health literacy is used, specifically the availability of services, existing support, financial resources, and so forth. To develop effective interventions to promote CHL in a specific context, a comprehensive understanding of the specific concept and a thorough situational analysis are essential.

### 1.2 The framework for situational analysis and intervention development

To date, no framework has been established for the promotion of CHL worldwide, whether in relation to general or specific interventions. It is therefore essential to employ alternative frameworks to inform the process of identification, coding, and analysis. In the Kenyan context, a variety of frameworks were employed, such as the socio-ecological model framework (37) and social psychological frameworks like the health belief model (38). One limitation of these frameworks is that they may be static or focus on a specific aspect of the process, such as the cognitive aspects, while neglecting other contextual factors, such as the political, social and healthcare environment. In response to the global need for a framework to guide health interventions, Green and Kreuter (39) developed a robust framework, the PRECEDE–PROCEED model. This model focuses on three core factors. *Predisposing factors*, are linked to the individual knowledge and attitudes; *enabling factors*, namely skills, resources, funding, stigma, and *reinforcing factors*, including social support. These factors can be influenced by *education* and *policy*, and in turn, can influence *behaviour* and the *environment*, thereby promoting *health* and ultimately leading to an improved *quality of life*. A thorough situational analysis of each factor and their interrelationships will facilitate the description of the concept and context, thus the identification of the necessary elements to tailor interventions and policy regulations to enforce it. The Precede–proceed model (40) is the most comprehensive and one of the most frequently employed approaches to the (holistic) promotion of health. The model was chosen for this analysis for five reasons: it incorporates the ecological model of health, maintains a population-centred approach, employs quality of life (rather than behaviour change) as the overarching outcome, and is firmly grounded in empirical evidence (41). It distinguishes between two evaluation tasks. The initial evaluation task, designated as PRECEDE, is conducted prior to the implementation of an intervention and comprises four distinct phases. Firstly, a social assessment of quality of life and health is conducted. This is followed by a behavioural and environmental assessment, the second phase. The third phase involves a detailed exploration of predisposing, enabling and reinforcing factors. The fourth and final phase addresses health education and policy formulation. In this final phase, measurable objectives and baselines can be specified. The second evaluation task, PROCEED, encompasses all the four phases but in reverse order, thereby providing a guideline for monitoring and continuous quality improvement. The Precede–proceed model is a frequently employed methodology for the qualitative investigation of intervention development (42). For the purposes of this study, we focused on the first evaluation task, PRECEDE.

## 2 Methodology

Given our objective of identifying the types of available evidence in the literature, examining the extant literature, clarifying the key concept of cancer health literacy in Kenya, and investigating the situation and factors influencing interventions to promote cancer health literacy in Kenya, a scoping review was deemed the most appropriate methodology (43). A scoping review was conducted in accordance with the five-step methodology proposed by Arksey and O'Malley (44): identification of research questions, identification of relevant studies, selection of studies, charting data, collation, summarisation, and reporting of results. Furthermore, the PRISMA-ScR checklist (45) guided reporting of this study.

*To identify relevant literature for this study*, a search was conducted on the following nine academic databases on two separate dates: October 27^th^, 2023, and February 5^th^, 2024. The databases were PubMed, Web of Science, CINAHL, APA PsycINFO, OpenDissertation, ERIC, Cochrane Library, African Journals Online, and African Index Medicus. A hand search of relevant journals and grey literature sources was conducted to identify all relevant studies. The search strategy was based on the PCC model recommended by the Joanna Briggs Institute (46). The keywords utilised in the titles and abstracts were “cancer” and “Kenya,” and “knowledge”, linked with their respective synonyms, and truncating words to encompass potential variations used in the literature.

- **Population**: (not specified, but focus on general population, patients, survivors, not health care providers).
- **Concept**:

° knowledge OR understanding OR awareness OR belief^*^ OR perception^*^ OR behaviour OR behavior OR practice^*^ OR experience^*^ OR skill^*^ OR competenc^*^ OR literacy OR competencies OR capabili^*^ OR abilit^*^ OR coping OR motivation,
° cancer or oncology or HPV.

- **Context:** Kenya OR Kenyan^*^.
- **+** **Time:** 2010 (adoption of the new constitution in Kenya highlighting devolution) till 2023.

The search strings for the individual databases are found in the Supplementary material 1.

The search was confined to literature published between the period between 01/01/2010 and 30/10/2023. The year 2010 was selected as the starting point due to its significance as the year in which the new Kenyan constitution was adopted, which included a decentralisation of health services. The evidence from CHLs across subnational levels may inform the development of targeted cancer control strategies in different counties and regions in Kenya. Any scientific study either published in a scientific journal or as a full report was included. Furthermore, the search was also limited to studies published in the English language, as it is the official language of Kenya.

Following the completion of the search on each database, the resulting bibliographic information was exported and imported into Rayyan.AI, a software tool designed for screening literature. Duplicates were identified through the utilisation of the automatic identification function within Rayyan, and subsequently verified and removed manually by one author (SH).

*Study selection process:* Two researchers (SH, LW) undertook an independent screening of titles and abstracts in Rayyan.AI in order to identify articles that met the inclusion and exclusion criteria and to remove irrelevant studies. Subsequently, SH and LW undertook a review of the full texts in order to identify the final set of articles. Any discrepancies regarding the inclusion of studies were resolved through discussion. In instances of persistent disagreement, a third reviewer (VOO) was consulted in order to reach a final decision. The studies included in this review met the following inclusion and exclusion criteria, as outlined in Table 1.

**Table 1:** Inclusion and exclusion criteria.

**Inclusion criteria**	**Exclusion criteria**
**Population**	
-General population -Cancer patients (or their family members)	-Focus on providers (not patients/general population) -Focus on training for providers
**Concept**	
-Focus on CHL-related aspects such as knowledge, awareness, information needs -Focus on cancer, HPV, or oncology	-No information about CHL relevant factors in result section (e.g., reports only on association between sociodemographic characteristics and screening uptake) -Focus primarily on other diseases than cancer (e.g., other non-communicable diseases)
**Context**	
-Conducted in Kenya -Starting from 2010 (after the new constitution of Kenya and thus more decentralisation till 2023) -Studies on situational features: barriers and facilitators for information or behaviour	-Studies or reviews that focus on multiple countries -Studies with data collection prior to 2010
**Study type**	
-Quantitative, qualitative and mixed-method studies providing information on CHL related aspects as objectives or findings	-Reviews -Only abstract available (e.g., conference abstract) -Study protocol Only report by a person about his experience as a short-term voluntary healthcare worker in Kenya

We did not critically appraise the quality of the included records as it is not an integral requirement for a scoping review (41).

*Data charting, analysis and results collating, summarising, and reporting. One* researcher (SH) undertook a thorough reading of the eligible articles several times to become familiar with the content. A coding scheme was developed by the researchers, and the data were extracted by SH and entered into an Excel spreadsheet with the following headings: author, year, location, aim, cancer, focal population, phase, sample, study design, method, single or multiple points in time, factors assessed, questionnaires, and interventions. A second author (LW) undertook a verification of the data entry. Study characteristics were summarised using numerical and thematic analyses. To analyse the articles' content qualitatively (47), two coding schemes were created based on the components of the selected frameworks (see Supplementary material 2 for the coding schemes). Firstly, data was analysed using the core constructs of the comprehensive health literacy model, comprising knowledge, motivation, competence, and the respective action words: find, understand, appraise, and apply (14). Secondly, data pertaining to the constructs of the precede–proceed model (40) was identified. These were policy, health education, predisposing factors, enabling factors, reinforcing factors, behaviour, environment, genetics, health, and quality of life. The data was then coded and analysed using MAXQDA 24, a software designed to support the analysis of qualitative data. In addition to the coding scheme, the authors employed two categorisations of the data, firstly based on the phase of the cancer care continuum, and secondly, based on intervention/no intervention. After extracting data pertaining to the different concepts and phases of the cancer care continuum separately, parent themes were identified deductively, subthemes identified inductively, and data was summarised.

## 3 Results

### 3.1 Empirical studies on cancer health literacy-related topics in Kenya

**Figure 1:**
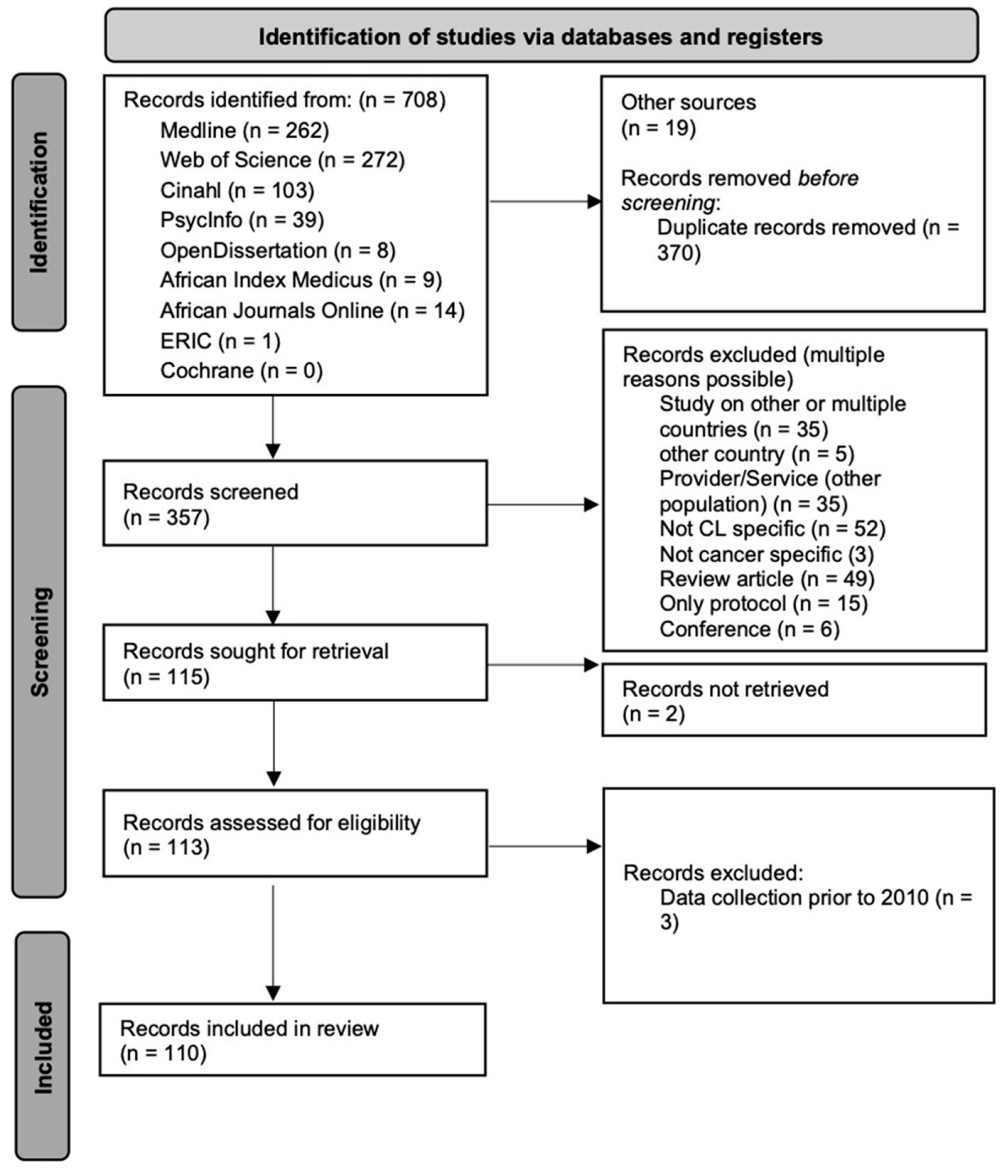
Prisma flow chart of studies on CHL in Kenya.

Studies addressing cancer health-literacy related concepts are common in Kenya, as more than 700 studies on CHL-related topics in Kenya were identified (Figure 1).

The initial search on six databases yielded 727 articles. Following the removal of duplicates, the titles and abstracts of 357 articles were reviewed, resulting in 115 articles for retrieval. Two articles could not be retrieved, and three articles were excluded after the full-text reading. A total of 110 articles met the eligibility criteria, of which 108 scientific articles and two research reports (5, 48), as illustrated in the Prisma flow chart (Figure 1). For further information, please refer to the sample description provided in Table 2 and the comprehensive list of articles in Table 3.

**Table 2:** Characteristics of studies included.

**Sample description**				**Sample description**			
**Indicator**	**Data**	**#**	**%**	**Indicator**	**Data**	**#**	**%**
**Characteristics of the article**					Uasin Gishu	11	10
Article type	Peer-reviewed article	108	98.18		Only one county	33	30
	Report	2	1.82		Nyanza	2	1.82
Study design	**Quantitative**	56	–		Not mentioned	1	0.91
	Secondary data analysis	7	12.5	Data provider	General population (both gender)	20	18.18
	Cross-sectional study	48	85.71		Women	58	52.73
	Longitudinal study	1	1.79		Men	10	9.09
	**Qualitative**	41	-		Youth	2	1.82
	Cross-sectional study	38	92.68		Providers	4	3.64
	Longitudinal study	3	7.32		Caregivers	7	6.36
	**Mixed Methods study**	13	-		Multiple	9	8.18
	Cross-sectional study	13	100	**Characteristics of the cancer**			
	Longitudinal study	0	0	Cancer type	Cervical	60	54.55
Observational/intervention	Observational study	96	87.27		Breast	18	16.36
	Intervention study	14	12.73		Prostate	7	6.36
Year	2010–2011	2	1.82		General	16	14.55
	2012–2013	6	5.45		Paediatric	4	3.64
	2014–2015	21	19.09		Kaposi	3	2.73
	2016–2017	16	14.54		Retinoblastoma	1	0.91
	2018–2019	20	18.18		Oesophageal	1	0.91
	2020–2021	17	15.45	Cancer phase	Unspecified	7	6.36
	2022–2023	28	25.45		Prevention	17	15.45
Location	Nairobi	19	17.27		Early detection	52	47.27
	Multiple counties	19	17.27		Treatment	31	28.18
	Country-wide	13	11.82		Survivorship	2	1.82
	Western	12	10.91		Palliative care	1	0.91

**Table 3:** Overview of studies included in the review.

**Author/year**	**Year**	**Title**	**Location**	**Cancer**	**Focal population**	**Phase**	**Methodology/ intervention**
Adewumi et al. (2019) (110)	2019	Female perspectives on male involvement in a human-papillomavirus-based cervical cancer-screening program in western Kenya	Western Kenya	Cervical cancer/HPV	women and community health volunteers (*N =* 604)	Screening	Qualitative
Adewumi et al. (2022) (111)	2022	Barriers and facilitators to cervical cancer screening in Western Kenya: A qualitative study	Migori County	Cervical cancer	Women (*N =* 24), providers (*N =* 12)	Screening	Qualitative
Alago and Awiti (2016) (105)	2016	Preferred breast cancer message sources and frames amongst western Kenyan women	Kisumu County	Breast cancer	Women (*N =* 393)	Prevention (vaccination)	Qualitative
American Cancer Society (48)	2016	Assessment of **knowledge, attitudes and practices** survey on cancer in selected regions of Kenya. Report of research findings	Kenya, across	Cancer	Both gender (patients, caregivers, expert stakeholders and health professionals)	Treatment	Mixed-methods
Antabe et al. (2020) (69)	2020	Utilization of breast cancer screening in Kenya: what are the determinants?	Kenya, across	Breast cancer	Women, representing all areas of the country (*n =* 14734)	Screening	Quantitative
Buchanan Lunsford et al. (2017) (112)	2017	Environmental and psychosocial barriers to and benefits of cervical cancer screening in Kenya	Nairobi and Nyanza	Cervical	Women and partners (*N =* 60/*N =* 40)	Screening	Qualitative
Busakhala et al. (2016) (113)	2016	Screening by clinical breast examination in Western Kenya: Who comes?	Western Kenya	Breast cancer	Women (*N =* 1978)	Screening	Quantitative
Busolo et al. (2023) (114)	2023	Kenyan youth's understanding of cancer and cancer risk: a qualitative study	Not mentioned	General	Youth (*N =* 53)	Prevention (vaccination)	Qualitative
Caren et al. (2020) (91)	2020	Experience of communication among cervical cancer patients in Kenya	Uasin Gishu County	Cervical cancer	Patients (*N =* 8) and caregivers (*N =* 8)	Treatment	Qualitative
Cheboi et al. (2023) (115)	2023	Health care seeking behaviors and perspective on indigenous palliative care among cancer patients in Kenya	Kenya, across, in urban and rural areas	General	Patients (*N =* 433)	Treatment	Quantitative
Choi et al. (2020) (9)	2020	A qualitative exploration of women's experiences with a community health volunteer-led cervical cancer **educational** module in Migori County, Kenya	Migori County	Cervical cancer	Women (*N =* 525 interviewed)	Screening	Qualitative intervention
Choi et al. (2022) (116)	2022	Uptake and correlates of cervical cancer screening among women attending a community-based multi-disease health campaign in Kenya	Kisumu city, informal settlement Obunga	Cervical cancer	Women (*N =* 749)	Screening	Quantitative
Choi et al. (2023) (72)	2023	The impact of text message reminders on uptake of cryotherapy among women testing positive for HPV in western Kenya	Migori County	Cervical cancer	Women (*N =* 2368)	Treatment	Qualitative intervention
Collier et al. (2022) (64)	2022	Telling the story of intersectional **stigma** in HIV-associated Kaposi's sarcoma in western Kenya: a convergent mixed-methods approach	Western Kenya	Kaposi sarcoma	People with HIV associated Kaposi's sarcoma (*N =* 117 and *N =* 88)	General	Mixed-methods
Daniel et al. (2023) (117)	2023	Delayed breast cancer presentation, diagnosis, and treatment in Kenya	Nairobi	Breast cancer	Women, female patients (*N =* 378)	Treatment	Mixed-methods
Duron et al. (2013) (8)	2013	Esophageal cancer awareness in Bomet district, Kenya	Bomet	Esophageal cancer	People at hospital (*N =* 81)	Prevention (vaccination)	Quantitative
Dutta et al. (2018) (70)	2018	Association between individual and intimate partner factors and cervical cancer screening in Kenya	Kenya, across	Cervical cancer	Women, responded to the cervical cancer screening and domestic violence questions (*N =* 3222)	Screening	Quantitative
Erena et al. (2020) (52)	2020	Prostate cancer awareness and screening practice among Kenyan men	Kenya, across	Prostate cancer	Men (*N =* 12803)	Screening	Quantitative
Friedman et al. (2014) (93)	2014	Preparing for human papillomavirus vaccine introduction in Kenya: implications from focus-group and interview discussions with caregivers and opinion leaders in Western Kenya	Western Kenya	Cervical cancer	General population (*N =* 56 in Focus group), key informants (*N =* 12)	Prevention (vaccination)	Qualitative
Gakunga et al. (2019) (118)	2019	Identifying Barriers and facilitators to breast cancer early detection and subsequent treatment engagement in Kenya: A qualitative approach	Nairobi county	Breast cancer	Women with and without diagnosis (6-11 people per FGD, 4 FGD)	Screening/detection	Qualitative
Gakunga et al. (2023) (107)	2023	Preferences for breast and cervical cancer screening among women and men in Kenya: Key considerations for designing implementation strategies to increase screening uptake	Six subcounties (a.o. Kiambu, Nairobi and Machakos counties)	Breast and cervical cancer	Patients: male (*N =* 429), female (*N =* 417)	Screening	Quantitative
Gatumo et al. (2018) (119)	2018	Women's knowledge and attitudes related to cervical cancer and cervical cancer screening in Isiolo and Tharaka Nithi counties, Kenya: a cross-sectional study	Isiolo county, Tharaka Nithi county	cervical cancer	Women (*N =* 451)	Screening	Quantitative
Gedleh et al. (2017) (65)	2017	“Where does it come from?” Experiences among survivors and parents of children with retinoblastoma in Kenya	Nairobi and Kikuyu	Retinoblastoma	Survivors and parents of children with retinoblastoma (*N =* 31)	Survivorship	Qualitative
Ginjupalli et al. (2022) (85)	2022	Developing a framework to describe stigma related to cervical cancer and HPV in western Kenya	Kisumu	Cervical cancer/HPV	Women living with HIV, HIV negative women, CHW, HC providers (*N =* 26)	General	Qualitative
Githaiga and Schwartz (2017) (89)	2017	“You have a swelling”: The language of cancer diagnosis and implications for cancer management in Kenya	Nairobi	Cancer	Women (*N =* 2)	Treatment	Qualitative
Githaiga et al. (2015) (49)	2015	Family cancer caregiving in urban Africa: interrogating the Kenyan model	Nairobi	cancer	caregivers (*N =* 20), interviews twice (*N =* 7), focus group (*N =* 13)	Treatment	Qualitative
Githaiga (2017) (120)	2017	When ‘chemo is failing' … ‘the illness is indigenous'. Therapeutic pluralism and reclaiming agency: family cancer caregivers' experiences in Nairobi	Nairobi	General	Caregivers (*N =* 20) family caregivers of patients with advanced cancer	Treatment	Qualitative
Gitonga et al. (2022) (25)	2022	Cervical cancer knowledge, awareness and related health behaviours amongst women of reproductive age in Kiambu County, Kenya: a cross-sectional study	Kiambu County	Cervical cancer	Women (*N =* 472), reproductive age	Screening	Quantitative
Henry et al. (2021) (84)	2021	Barriers to communicating a cancer diagnosis to patients in a low- to middle-income context	Kenya, across	Cancer	Health care workers: 114 professionals	Treatment	Qualitative
Huchko et al. (2019) (121)	2019	‘I'm here to save my life': a qualitative study of experiences navigating a cryotherapy referral system for human papillomavirus-positive women in western Kenya	Migori County	Cervical cancer/HPV	Female patients (*N =* 273), (women undergoing cryotherapy)	Treatment	Qualitative
Isaacson et al. (2023) (122)	2023	A qualitative exploration of barriers to treatment among HPV-positive women in a cervical cancer screening study in Western Kenya	Migori County	HPV/cervical cancer	Women HPV positive who did not attend no-cost cryotherapy treatment (*N =* 84)	Screening and treatment	Qualitative
Kailemia et al. (2023) (31)	2023	Intersection of social determinants of symptomatic breast cancer presentation in a rural setting: A critical ethnographic study	Meru County	Breast cancer	Women (*N =* 12), disclosure recipients (*N =* 23)	Treatment	Qualitative
Kangethe et al. (2022) (123)	2022	Utilisation of cervical cancer screening among women living with HIV at Kenya's national referral hospital	Nairobi (Kenyatta National Hospital)	Cervical cancer	Women, women living with HIV (*N =* 305 + FGD)	Screening	Mixed-methods
Kangmennaang et al. (2018) (16)	2018	The next Sub Saharan African epidemic? A case study of the determinants of cervical cancer knowledge and screening in Kenya	Kenya, across	Cervical cancer	Women reproductive age (*N =* 11,138/10,333)	Screening	Quantitative
Kangwana et al. (2022) (124)	2022	Barriers to cryotherapy treatment services for precancerous cervical lesions among women in Western Kenya	Migosi Sub County	Cervical cancer	Women, reproductive age (*N =* 60)	Treatment	Quantitative
Kassaman et al. (2022) (22)	2022	Fear, faith and finances: health literacy experiences of English and Swahili speaking women newly diagnosed with breast and cervical cancer	Nairobi, Central Kenya, (2 hospitals in Nairobi)	Breast and cervical cancer	Women, patients, Newly diagnosed women (*N =* 18)	Treatment	Qualitative
Kemper et al. (2019) (125)	2019	Geographic and individual correlates of cervical cancer screening among HIV-infected women attending HIV Care and Treatment Programs in Kenya	Kenya, across	Cervical cancer	Women (*N =* 3,007)	Screening	Quantitative
Kemper et al. (2022) (126)	2022	Correlates of cervical cancer screening among women living with HIV in Kenya: A cross-sectional study	Kenya, across	Cervical cancer	Women (*N =* 3007)	Screening	Quantitative
Kinyao and Kishoylan (2018) (57)	2018	Attitude, perceived risk and intention to screen for prostate cancer by adult men in Kasikeu Sub Location, Makueni County, Kenya	Makueni County (rural Kenya)	Prostate cancer	Men (*N =* 155)	Screening	Quantitative
Kisiangani et al. (2018) (10)	2018	Determinants of breast cancer early detection for cues to expanded control and care: the lived experiences among women from Western Kenya	Kakamega	Breast cancer	Adult participants from rural and urban settings (6–10 members per FG, 8 FG)	Treatment	Qualitative
Kisuya et al. (2015) (79)	2015	Impact of an educational intervention on breast cancer knowledge in western Kenya	Western Kenya: Kakamega (Turbo), Nandi (Mosoriot) and Bungoma (Kapsokwony)	Breast cancer	Women (*N =* 532)	Screening	Quantitative intervention study
Kivuti-Bitok et al. (2012) (127)	2012	Self-reported use of internet by cervical cancer clients in two National Referral Hospitals in Kenya	Hospitals: Nairobi/Eldoret	Cervical cancer	Cervical patients (*N =* 199)	Treatment	Quantitative
Kivuti-Bitok et al. (2013) (128)	2013	An exploration of opportunities and challenges facing cervical cancer managers in Kenya	Multiple, provincial and national hospital	Cervical cancer	Provider (21) nurse managers and twelve (12) medical doctors	Treatment	Qualitative
Kolek et al. (2022) (129)	2022	Impact of parental knowledge and beliefs on HPV vaccine hesitancy in Kenya-findings and implications	Nairobi (Kenyatta National Hospital)	Cervical cancer/HPV	Parents of children to be vaccinated (*N =* 195)	Prevention (vaccination)	Quantitative
Lee et al. (2018) (26)	2018	In their own words: a qualitative study of Kenyan breast cancer survivors' knowledge, **experience**s, and attitudes regarding breast cancer genetics.	Nairobi	Breast cancer	Women breast cancer survivor (*N =* 21 in Focus groups)	Survivorship	Qualitative
Lehmann et al. (2020) (5)	2020	Economic and social consequences of cancer in case studies of selected households	Multiple Nakuru, Kisumu	Cancer	Households (*N =* 8 households, 16 participants)	Treatment	Qualitative
Libes et al. (2015) (58)	2015	Risk factors for abandonment of Wilms tumor therapy in Kenya	Hospitals: KNH and Moi hospital	Paediatric cancer	Patients: *N =* 136 registered patients (parents of patients)	Treatment	Quantitative
Mabeya et al. (2018) (130)	2018	Uptake of three doses of HPV vaccine by primary school girls in Eldoret, Kenya; a prospective cohort study in a malaria endemic setting	Eldoret	Cervical cancer/HPV	Girls (*N =* 3, 083)	Prevention (vaccination)	Quantitative
Mabeya et al. (2021) (131)	2021	Mothers of adolescent girls and Human Papilloma Virus (HPV) vaccination in Western Kenya	Eldoret, Uasin Gishu County	Cervical cancer/HPV	Mothers, accompanying their daughters to gynecological and adolescents clinics	Prevention (vaccination)	Mixed-methods
Makau-Barasa et al. (2018) (30)	2018	Improving access to cancer testing and treatment in Kenya	Nairobi	Cancer	Oncology clinicians (*N =* 7), support and advocacy leaders (*N =* 7)	General	Qualitative
Masika et al. (2015) (132)	2015	Knowledge on HPV vaccine and cervical cancer facilitates vaccine acceptability among school teachers in Kitui county, Kenya	Kitui	HPV/cervical cancer	Teachers (*N =* 339)	Prevention (vaccination)	Mixed-methods
Mbugua et al. (2021) (53)	2021	Prostate cancer awareness and screening among men in a rural community in Kenya: a cross-sectional study	Gatundu North and Kiambu Sub-counties	Prostate	Men aged 40–69 (*N =* 576), 44 men in FGD	Screening	Mixed-methods
Mbugua et al. (2022) (29)	2022	Effectiveness of a community health worker-led intervention on knowledge, perception, and prostate cancer screening among men in rural Kenya	Multiple: Gatundu North subcounty, Kiambu County (control)	Prostate	Men aged 40–69 years (*N =* 280/287)	Screening	Quantitative intervention study
Mburu et al. (2019) (133)	2019	Knowledge of cervical cancer and acceptability of prevention strategies among HPV-vaccinated and nonvaccinated adolescents in Eldoret, Kenya	Eldoret	Cervical cancer	Women (*N =* 180, 120 unvaccinated adolescent women, 60 vaccinated adolescent women)	Prevention (vaccination)	Quantitative
McMahon et al. (2022) (63)	2022	Understanding diagnostic delays for Kaposi sarcoma in Kenya: a qualitative study	Western Kenya	Kaposi sarcoma	Newly diagnosed Kaposi Sarcoma patients (*N =* 30)	Treatment	Qualitative
McMahon et al. (2022) (62)	2022	Barriers and facilitators to chemotherapy initiation and adherence for patients with HIV-associated Kaposi's sarcoma in Kenya: a qualitative study	Western Kenya	Kaposi sarcoma	Newly diagnosed Kaposi Sarcoma patients (*N =* 57)	Treatment	Qualitative
Morema et al. (2014) (134)	2014	Determinants of cervical screening services uptake among 18–49 year old women seeking services at the Jaramogi Oginga Odinga Teaching and Referral Hospital, Kisumu, Kenya	Kisumu, Nyanza	Cervical cancer	Women of child-bearing age at Jaramogi Oginga Odinga TRH (*N =* 424)	Screening	Quantitative
Mostert et al. (2014) (135)	2014	Two overlooked contributors to abandonment of childhood cancer treatment in Kenya: Parents' social network and **experience**s with hospital retention policies	Eldoret	Paediatric/childhood cancer	Parents of childhood cancer patients (*N =* 98)	Treatment	Qualitative
Muchiri et al. (2021) (73)	2021	Narrative persuasion: Effects of narrative message frame on intention to screening for cervical cancer among women in agricultural sector, Kiambu County, Kenya	Kiambu County	Cervical cancer	Participants (female) (*N =* 378 and *N =* 344)	Screening	Quantitative intervention study
Muchiri et al. (2021) (74)	2021	Narrative Persuasion: Moderating effects of character identification on relationship between message format and intention to screen for cervical cancer among women in agricultural sector in Kiambu County, Kenya.	Kiambu County	Cervical cancer	Participants (female) (*N =* 378 and *N =* 344)	Screening	Quantitative intervention study
Muinde et al. (2020) (76)	2020	Effect of a community health worker intervention on uptake of breast cancer screening services among women of reproductive age in Kitui county, Kenya	Kitui East, Mwingi West	Cervical cancer	Women (*N =* 402/409)	Screening	Quantitative intervention study
Muinde et al. (2021) (75)	2021	Effect of a community health worker based health promotion intervention on uptake of cervical cancer screening services among women of reproductive age in Kitui County, Kenya	Kitui East, Mwingi West	Cervical cancer	Women (*N =* 402/409)	Screening	Quantitative intervention study
Muthike et al. (2015) (136)	2015	Nutritional knowledge and dietary diversity of cancer patients at the Cancer Treatment Centre, Kenyatta National Hospital, Kenya	Nairobi (Kenyatta National Hospital)	Cancer	Patients (*N =* 132)	Treatment	Quantitative
Muthoni et al. (2010) (137)	2010	An exploration of rural and urban Kenyan women's knowledge and attitudes regarding breast cancer and breast cancer early detection measures	Kiambu District, Kamba Machokos District (Kikuyu)	Breast cancer	Women: low- and middle-income rural and urban Kenyan women, either 20–35 years or 36–60 years (8 FGD, each 6–7 participants)	Screening	Qualitative
Mutua et al. (2017) (55)	2017	Cultural factors associated with the intent to be screened for prostate cancer among adult men in a rural Kenyan community	Kasikeu, Makueni County	Prostate	Men (*N =* 155)	Screening	Quantitative
Muturi et al. (2020) (83)	2020	eHealth literacy and the motivators for HPV prevention among young adults in Kenya	Multiple (2 private and 2 public universities)	Cervical cancer	Youth (*N =* 472)	Prevention (vaccination)	Quantitative
Mwangi et al. (2018) (92)	2018	Quality of life for family caregivers to cancer patients in Kenyatta National Hospital Nairobi city county, Kenya	Nairobi (Kenyatta National Hospital)	Cancer	Family caregivers (*N =* 164)	Treatment	Quantitative
Mwangi et al. (2022) (138)	2022	Factors effecting quality of life for family caregivers of cancer patients in Kenya	Nairobi (Kenyatta Teaching, Transferral and Research Hospital)	Cancer	Caregivers: 164 family caregivers of cancer patients	Treatment	Quantitative
Mwenda et al. (2022) (80)	2022	Breast health awareness campaign and screening pilot in a Kenyan County: Findings and lessons	Nyeri County	Breast cancer	Women (*N =* 1,813)	Screening	Qualitative intervention
Naanyu et al. (2015) (88)	2015	Lay perceptions of breast cancer in Western Kenya	Western Kenya: Uasin Gishu County, Nandi County, Mount Elgon	Breast cancer	Both gender: men and women (*N =* 1,335)	General	Mixed-methods
Ndetei et al. (2018) (87)	2018	Psychological well-being and social functioning across the cancer stages: implications for palliative care	Nairobi	General	Patients (*N =* 389)	General	Quantitative
Ng'ang'a et al. (2018) (139)	2018	Predictors of cervical cancer screening among Kenyan women: results of a nested case-control study in a nationally representative survey	Kenya, across	Cervical cancer	Women (*N =* 1,180)	Screening	Quantitative
Ngugi et al. (2012) (140)	2012	Factors affecting uptake of cervical cancer early detection measures among women in Thika, Kenya	Thika, Kenya	Cervical cancer	Women of the general population (*N =* 50)	Screening	Mixed-methods
Ngune et al. (2020) (68)	2020	Biopsychosocial risk factors and knowledge of cervical cancer among young women: A case study from Kenya to inform HPV prevention in Sub-Saharan Africa	Kenya, across	Cervical cancer	Women 15–24 years (*N =* 5,398)	Prevention (vaccination)	Quantitative
Ngutu et al. (2015) (141)	2015	Exploring the barriers to health care and psychosocial challenges in cervical cancer management in Kenya	Nairobi	Cervical cancer	Patient, female, women living with cervical cancer (*N =* 18)	Treatment	Qualitative
Njuguna et al. (2014) (60)	2014	Abandonment of childhood cancer treatment in Western Kenya	Eldoret, Moi Teaching and Referral Hospital (MTRH)	Paediatric cancer	Parents of children with cancer	Treatment	Qualitative
Njuguna et al. (2015) (61)	2015	Parental experiences of childhood cancer treatment in Kenya	Eldoret, Moi Teaching and Referral Hospital	Paediatric cancer	Parents of childhood cancer patients (*N =* 75)	Treatment	Quantitative
Njuguna et al. (2021) (142)	2021	Knowledge, attitude and practice of main stakeholders towards human papilloma virus infection and vaccination in Mombasa and Tana-river counties in Kenya: a qualitative study	Mombasa county, tana-river counties	Cervical cancer/HPV	Children, parents, head teachers, community leaders, health workers	Prevention (vaccination)	Qualitative
Nmoh (2019) (143)	2019	Cancer management in Kenya—awareness and the struggles patients face to access treatment, care and support	Kisumu	Cancer	Cancer patients, key informants, women	Treatment	Mixed-methods
Nyawira Githaiga and Swartz (2017) (66)	2017	Socio-cultural contexts of end-of-life conversations and decisions: bereaved family cancer caregivers' retrospective co-constructions	Nairobi	Cancer	Women cancer caregivers 4 FG (*N =* 13 participants)	Palliative	Qualitative
Oketch et al. (2019) (144)	2019	Perspectives of women participating in a cervical cancer screening campaign with community-based HPV self-sampling in rural western Kenya: a qualitative study	Migori County	Cervical cancer	Women (*N =* 120)	Screening	Qualitative
Okyere et al. (2023) (56)	2023	Prostate cancer screening uptake in Kenya: An analysis of the demographic and health survey	Kenya, across	Prostate cancer	Men (*N =* 7,923)—who have ever heard of prostate cancer	Screening	Quantitative
Omolo et al. (2022) (145)	2022	Psychological factors associated with the uptake of screening services for early detection of cancer among clients visiting Masinga level four hospital outpatient department, Masinga Sub County, Machakos County, Kenya	Machakos County	Cancer	Mixed (*N =* 158), 9 Focus group discussion	Screening	Mixed-methods
Omondi et al. (2022) (146)	2022	Factors influencing cervical cancer screening among pregnant women in <city>Nairobi </city>, Kenya	Nairobi	Cervical cancer	Women, pregnant (*N =* 107)	Screening	Quantitative
Opondo et al. (2022) (54)	2022	Effect of perceived self-vulnerability on prostate cancer screening uptake and associated factors: a cross-sectional study of public health facilities in Western Kenya	Kisumu County	Prostate	Male health workers (*N =* 197)	Screening	Quantitative
Orang'o et al. (2016) (147)	2016	Factors associated with uptake of visual inspection with acetic acid (*via*) for cervical cancer screening in Western Kenya	Western Kenya	Cervical cancer	Women (*N =* 2505)	Screening	Quantitative
Oriko (2020) (148)	2020	Men's knowledge and perceptions of cervical cancer: Influence upon increase in cervical cancer screening in rural Kenya	Kendubay	Cervical cancer	Men (*N =* 15)	Screening	Qualitative
Page et al. (2020) (149)	2020	Systems-level barriers to treatment in a cervical cancer prevention program in Kenya: Several observational studies	Western Kenya	Cervical cancer	Provider (*N =* 16)	Treatment	Quantitative
Ragan et al. (2018) (150)	2018	Perspectives of screening-eligible women and male partners on benefits of and barriers to treatment for precancerous lesions and cervical cancer in Kenya	Multiple: Nairobi, Nyanza	Cervical cancer	Women (*N =* 60), Male partners (*N =* 40)	Screening	Qualitative
Rositch et al. (2012) (151)	2012	Knowledge and acceptability of pap smears, self-sampling and HPV vaccination among adult women in Kenya	Nairobi	HPV/cervical cancer	Women (*N =* 409)	Prevention (vaccination)	Qualitative
Rosser et al. (2014) (152)	2014	Men's knowledge and attitudes about cervical cancer screening in Kenya	Nyanza Province	Cervical cancer	Men (*N =* 110)	Screening	Quantitative
Rosser et al. (2015) (77)	2015	Changing knowledge, attitudes, and behaviors regarding cervical cancer screening: The effects of an educational intervention in rural Kenya	Suba, Mbita	Cervical cancer	Women attending health facilities (*N =* 207/212)	Screening	Quantitative intervention study
Rosser et al. (2015) (153)	2015	Knowledge about cervical cancer screening and perception of risk among women attending outpatient clinics in rural Kenya	Western Kenya (health facilities)	Cervical cancer	Women, non-pregnant aged 23–64 years who attended one of 11 western Kenyan health facilities	Screening	Quantitative
Rosser et al. (2016) (154)	2016	Cervical cancer stigma in rural Kenya: what does HIV have to do with it?	Mbita, Suba	Cervical cancer	Women (*N =* 419)	General	Quantitative
Rosser, Hamisi et al. (2015) (155)	2015	Barriers to cervical cancer screening in rural Kenya: perspectives from a provider survey	Suba, Mbita	Cervical cancer	Staff members (*N =* 106)	Screening	Quantitative
Rosser, Njoroge et al. (2015) (86)	2015	Cervical cancer screening knowledge and behavior among women attending an urban HIV clinic in Western Kenya	Nyanza province	Cervical cancer	Women, HIV patients (*N =* 106)	Screening	Quantitative
Sayed et al. (2016) (81)	2016	Breast camps for awareness and early diagnosis of breast cancer in countries with limited resources: a multidisciplinary model from Kenya	multiple: Hospital (3 different: Mombasa, Bomet, Kisii)	Breast cancer	Women (*N =* 1,094)	Screening	Quantitative intervention study
Sayed et al. (2019) (156)	2019	Breast Cancer knowledge, perceptions and practices in a rural Community in Coastal Kenya	Kaloleni, Kilifi County, Kenya	Breast cancer	Multiple: women and male heads of household	General	Mixed-methods
Shaikh et al. (2022) (82)	2022	Supporting Kenyan women with advanced breast cancer through a network and assessing their needs and quality of life	Kenya, across	breast cancer	Cancer patients, metastatic breast cancer (*N =* 114; mean age 51.4)	Treatment	Qualitative intervention
Stocks et al. (2022) (157)	2022	Mobile phone ownership and use among women screening for cervical cancer in a community-based setting in Western Kenya: Observational study.	Migori County	Cervical cancer	Women (*N =* 3,299)	Screening	Quantitative
Sudenga et al. (2013) (158)	2013	Knowledge, attitudes, practices, and perceived risk of cervical cancer among Kenyan women: brief report	Kisumu	Cervical cancer	Women (*N =* 388) reproductive health service	Screening	Quantitative
Tiruneh et al. (2017) (71)	2017	Individual-level and community-level determinants of cervical cancer screening among Kenyan women: a multilevel analysis of a Nationwide survey	Multiple: Centra, Nyanza and Nairobi regions	Cervical cancer	Women, married, reproductive age (15–49 years) (*N =* 9,016)	Screening	Quantitative
Vermandere et al. (2014) (50)	2014	Determinants of acceptance and subsequent uptake of the HPV vaccine in a cohort in Eldoret, Kenya	Eldoret	Cervical cancer/hpv	Women: mothers of children (*N =* 287 and *N =* 256)	Prevention (vaccination)	Quantitative intervention study
Vermandere et al. (2015) (51)	2015	Implementation of an HPV vaccination program in Eldoret, Kenya: results from a qualitative assessment by key stakeholders	Eldoret	HPV/cervical cancer	Teachers and fathers (*N =* 67)	Prevention (vaccination)	Qualitative
Vermandere et al. (2016) (78)	2016	Uptake of the human papillomavirus vaccine in Kenya: testing the health belief model through pathway modeling on cohort data	Eldoret	Cervical cancer/hpv	Mothers of school girls (*N =* 255)	Prevention (vaccination)	Quantitative intervention study
Wachira et al. (2014) (159)	2014	Barriers to uptake of breast cancer screening in Kenya	Mosoriot, Turbo, Kapsokwony	Breast cancer	Community members (18 years and above) (*N =* 733)	Screening	Quantitative
Wachira et al. (2017) (160)	2017	Refining a questionnaire to assess breast cancer knowledge and barriers to screening in Kenya: Psychometric assessment of the BCAM	Western Kenya	Breast cancer	women (*N =* 48 in FGD; *N =* 1,061 in survey)	Screening	Mixed-methods
Wamburu et al. (2016) (161)	2016	Association between stage at diagnosis and knowledge on cervical cancer among patients in a Kenyan tertiary hospital: a cross-sectional study	Nairobi (Kenyatta National Hospital)	Cervical cancer	Female patients (*N =* 361), (women diagnosed with cervical cancer)	Treatment	Quantitative
Watson-Jones et al. (2015) (162)	2015	Access and **Attitudes** to HPV Vaccination amongst Hard-To-Reach Populations in Kenya	Kajiado County/Korogocho informal settlement	Cervical cancer/HPV	Mixed focus group discussions (*N =* 14) and semi-structured interviews (*N =* 28) with health workers, parents, youth, and community and religious leaders	Prevention (vaccination)	Qualitative
Were et al. (2011) (163)	2011	Perceptions of risk and barriers to cervical cancer screening at Moi Teaching and Referral Hospital (MTRH), Eldoret, Kenya	Eldoret, Moi Teaching and Referral Hospital	Cervical cancer	Women, non-pregnant (*N =* 219)	Screening	Quantitative

More than half of the articles (51%) employed exclusively quantitative methods, while 11 % were mixed-method studies. Also, most studies were observational in nature, with only 14 studies describing and evaluating interventions. Additionally, the majority of studies employed data collection at a single point in time, for example in cross-sectional studies. Only four studies embraced a longitudinal study design and thus qualify for reporting the development of cancer health literacy over time (22, 49–51).

**Figure 2:**
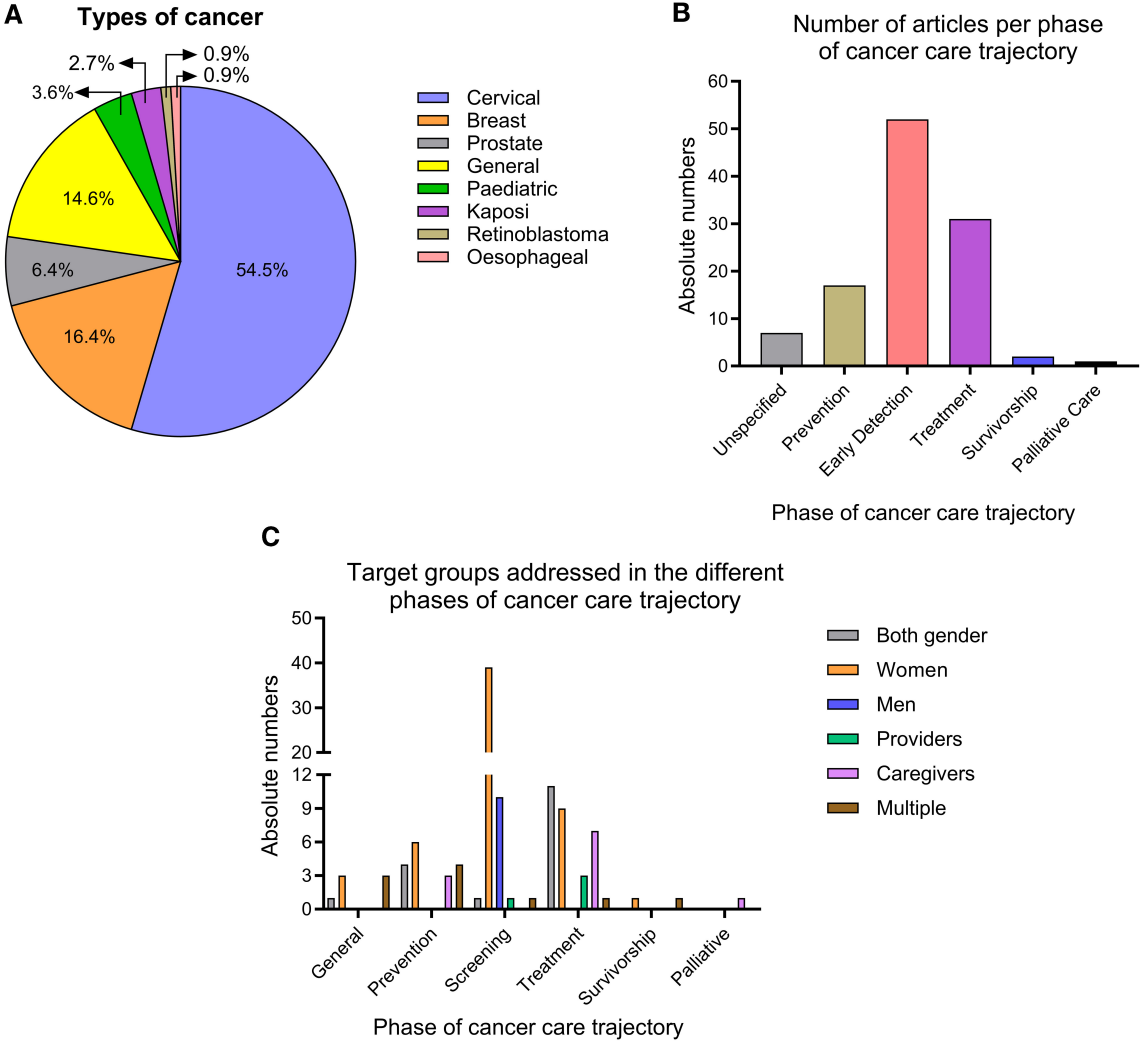
Specific characteristics of the research on CHL in Kenya. **(A)** Overview of cancer types addressed. **(B)** Phases of cancer care addressed. **(C)** Target group addressed in different phases of the cancer care.

With regard to the specific type of cancer type and the phase of cancer care, the majority of the studies (54.5%) focused on cervical cancer, while 16.4% addressed breast cancer and 14.5 % cancer in general. Only seven studies addressed prostate cancer (29, 52–57) and four focused on paediatric cancer (58–61). Notwithstanding its status as one of the five cancers with the highest incidence in Kenya (2), oesophageal sarcoma was only addressed in only a single study (8). Kaposi sarcoma was addressed in three studies (62–64). Retinoblastoma was also only addressed in one study (Figure 2A).

Regarding the cancer phase it was found that the majority of studies (46.4%) focused on the early detection & screening (also known as early diagnosis), followed by treatment (28.2%) and prevention (15.5%). Studies on survivorship (26, 65) or palliative care (66) were rare (Figure 2B).

The data revealed a gender disparity in CHL research. Women were interviewed with greater frequency (in 52.7% of all articles) than men (9.1%), particularly in studies pertaining to the prevention and screening of cancer. Conversely, studies on treatment and general cancer-related topics tend to adopt a more inclusive approach, encompassing both genders. There is a paucity of studies that incorporate multiple perspectives (only 8.2%), including those of patients and caregivers (Figure 2C).

**Figure 3:**
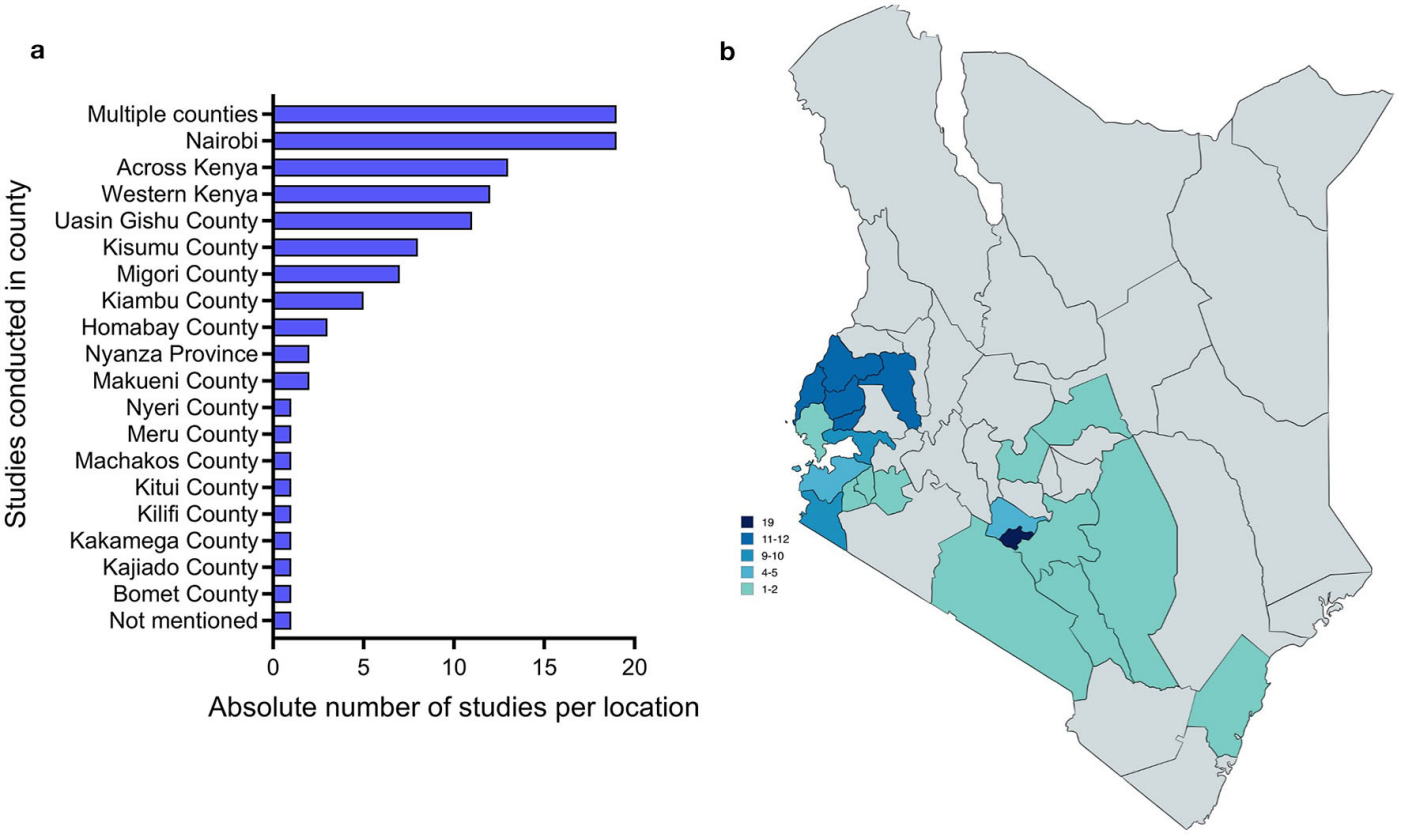
Geographical distribution of studies on cancer care in Kenya in numbers **(a)** and visualized on a map **(b)** (designed with mapchart).

Since the enactment of the new Kenyan Constitution in 2010, a number of studies have been conducted in various regions and counties throughout Kenya, representing a significant proportion of the total number of counties. The majority of these studies were conducted in the locations where the three long-standing Level 6 teaching and referral hospitals with cancer treatment facilities are situated, namely Nairobi and the Moi Teaching and Referral Hospital in Eldoret, Uasin Gishu County. Twelve studies have been carried out in the counties of the former province of “Western Kenya”, which has a high population density (67). While data from other regions is lacking, it seems reasonable to assume that the challenges identified in studies from remote areas may be similar in other remote areas. Seven out of the 14 nationwide studies employed secondary data analysis based on the 2014 Demographic and Health Survey (16, 52, 56, 68–71), which included questions on cancer awareness (Figure 3).

**Figure 4:**
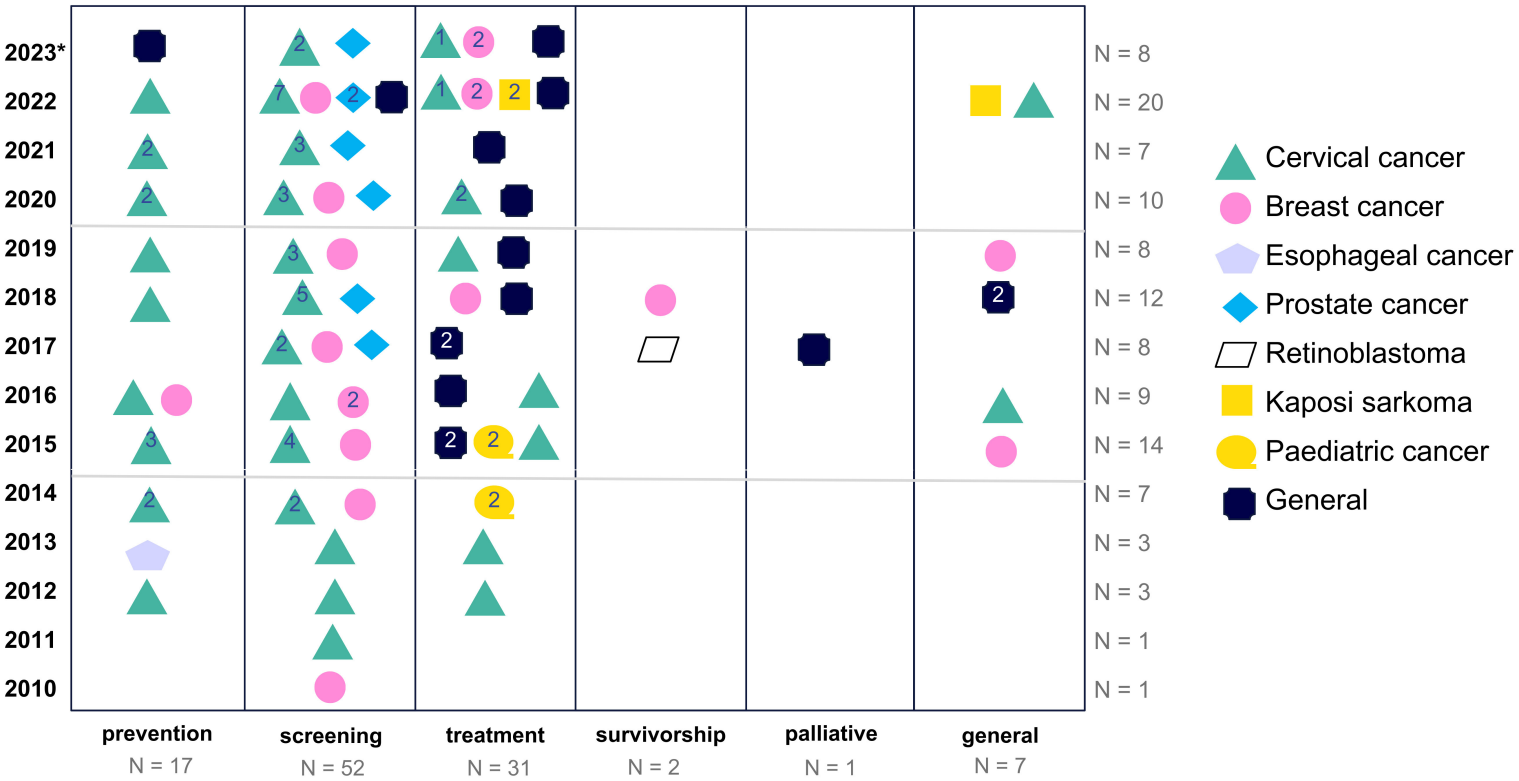
Historical trend of the focus on cancer care phases (^*^2023 only includes articles of the first 10 months).

A review of the historical development of research focus and cancer types reveals the emergence of distinct patterns of focus at different stages of the cancer care continuum. The focus on screening has remained consistently high throughout the years, with an increase in the absolute number of studies to eleven in 2022. Nevertheless, studies concentrating on the prevention of cancer have declined in recent years. It is noteworthy that there has been no mention of palliative care in the last 5 years (Figure 4).

The study design employed to investigate the various phases yielded clear trends. The majority of studies focusing on the prevention and screening phase employed quantitative research methods, whereas qualitative studies were more prevalent in the investigation of CHL concepts related to the treatment phase.

**Figure 5:**
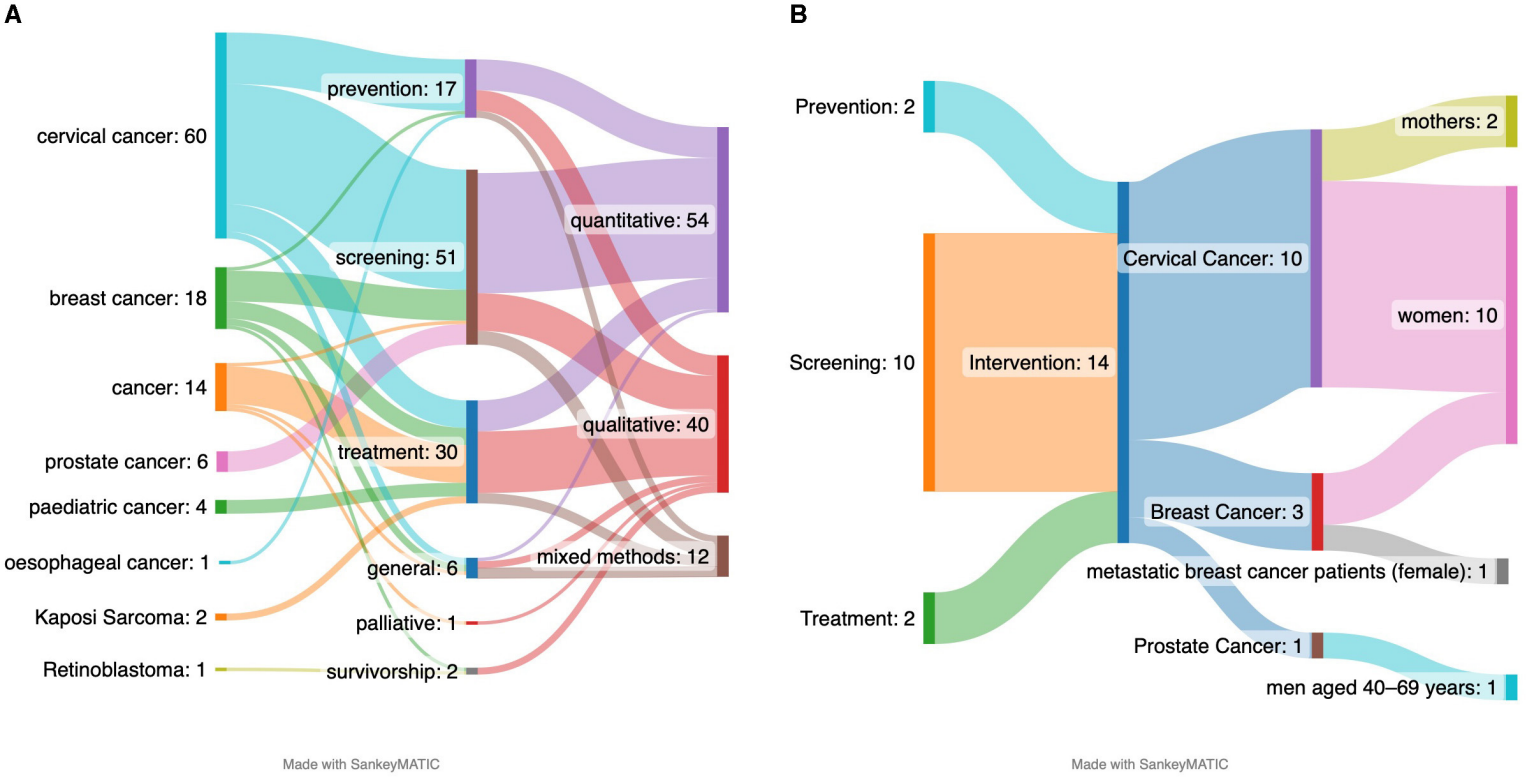
Observations of cancer type with phase and methods **(A)** and the intervention and target groups **(B)**.

The extant empirical evidence on cancer education programmes is, on the whole, insufficient. Of the 110 studies reviewed, only 14 (12.7%) reported on interventions. Of the 14 identified interventions, nine were focused on cervical cancer (9, 50, 72–78), four on breast cancer (79–82), and one on prostate cancer (29). Most interventions addressed the screening phase, with two focusing on prevention (50, 78) and two on treatment (72, 82). The interventions addressing the treatment phase both employed technology. Choi et al. (72) aimed at improving the link between screening and treatment through mHealth offers, specifically using text messaging. Similarly, Shaikh et al. (82) developed a web-based portal for patients with metastatic breast cancer (Figure 5).

### 3.2 Clarifying the concept of cancer health literacy

A mere nine articles employed the term “health literacy.” Two studies addressed the issue of prevention (68, 83), two studies were linked to screening (16, 27), and five studied were linked to treatment (5, 22, 62, 63, 84). The remaining studies concentrated on (the deficiencies in) knowledge, awareness, or (health) information among the general population or specific patient groups. However, a thorough analysis of the studies referring to the processes of finding, understanding, appraising, or applying (and their synonyms) of the information, knowledge, awareness reveals that the “operational use of information” is seldom included. Please refer to Supplementary material 3 for a detailed overview of all aspects related to health literacy. The tables below present a qualitative thematic summary of the primary and secondary themes.

**General cancer:** The category of “general cancer” encompassed seven articles, three of which address the issue of stigma (64, 85, 86), one article focuses on access (27), one article examines psychological well-being and social functioning (87) and two additional articles address knowledge, perceptions, and practices (81, 88) related to cancer testing and treatment throughout the cancer care continuum. It is notable that none of the articles utilized the terms “health literacy”, “cancer literacy”, or “cancer health literacy”, but they specifically referred to knowledge about stigma.

**Prevention:** The prevention category comprised 17 studies that focused on general awareness raising and vaccination. Two articles employed the specific terminology of health literacy. In their study, Ngune et al. explored the knowledge of cervical cancer among young women. As posited by Ngune et al. (68) “health literacy determined by access to mass media, such as radio, television and newspaper, has a significant impact on knowledge levels of cervical cancer. The lower the engagement and access, the higher the odds for low awareness.” The study findings indicate that low awareness is particularly prevalent among women of a lower economic status. Similarly, Muturi et al. (83) investigated the association between eHealth literacy, as measured by Norman and Skinner's eHEALS scale, and HPV knowledge, and other factors related to HPV infection prevention, among young people. They found that young people with higher eHealth literacy level exhibited more positive attitudes and behaviours in relation to prevention, for example seeking health information more frequently and demonstrating greater HPV knowledge etc. In this phase of the cancer care continuum, knowledge, awareness, and cancer information play a significant role, particularly, in the context of cervical cancer. Although there is a slight tendency to use the term “awareness” to refer to whether a person is familiar with a specific term or has heard of it. The terms “knowledge”, “awareness”, and “information” are frequently employed interchangeably (see Supplementary material 3 and Table 4 Phase 1).

**Table 4:** Conceptualization of cancer health literacy.

**Dimension**		**Activities**	
**Factor**	**Specifications**	**Dimension**	**Specification**
**Phase 1: prevention**			
**Knowledge**	Human body: cervix, the normal appearance of a breast, biological knowledge; Cancer and specific types of cancer, e.g., cervical cancer, oesophageal cancer, breast cancer; Symptoms of cancer and side-effects; Cause of cancer: Human Papilloma Virus, transmission, link between HPV and cervical cancer; Risk factors: early sexual debut, smoking, having multiple sexual partners, minimal symptomatic in an early stage, HPV infection, geography; Prevention modalities: Vaccination, pap smear test (goal); Services: regulation: HPV vaccination for target group (girls aged 10 and above), service for free, where and when available ; Treatment modalities: chemotherapy, radiation, and surgery; Characteristic of the information: Accurate/right/credible/proper; Misinformation	**Find**	Information, medical attention/treatment, permission, online-health seeking
**Motivation**	Beliefs: seriousness of threat, hesitancy, acceptability, willingness to vaccinate	**Understand**	General: public health information; The disease; Severity/gravity of cancer, the importance of HPV vaccination
**Competence**	Functional: seek/read information; Decision-related/evaluate/appraise (e.g., threats, coping); communication	**Appraise**	Make informed decisions, regarding preventive breast cancer measures; Acceptance of HPV vaccine; Treats and copings; Evaluate online health information
		**Apply**	Use online health information
**Phase 2: early detection**			
**Knowledge**	**Health concern related and or process related;** Health status: anatomy, healthy lifestyle, importance of check-ups; disease status: risk factors, symptoms, signs, e.g., of breast cancer or prostate cancer; HPV: transmission, progression from HPV to cervical cancer, HPV testing, fertility vaccination; Cervical cancer: definition, progression, symptoms, treatment, statistics; Prevention, process to seek treatment, treatment options; Screening: (procedures/tests, benefits of early detection; Self and perceived choices; Partners support myths	**Find**	Seek and access information (also through media, radio); Seek permission to attend services; Seek information by partner (not done)
**Motivation**	To initiate preventive behaviour/to go for screening, Fear of cancer hampers/motivates going for screening	**Understand**	HPV transmission, Importance of treatment; Possibility of re-infection; Results
**Competence**	Perform self-examination, Accept and complete HPV self-sampling; Access information, seek services, respond to questions	**Appraise**	Symptoms; Preventive behaviour and accept screening; Accept educational message; Accept health service, screening and screening methods; Different information
		**Apply**	
**Phase 3: treatment**			
**Knowledge**	Nature of disease: signs, symptoms etc.; Benefits of screening, early detection; Accessing treatment: navigating hospital, Alternative places to receive treatment; Results; Treatment modalities: cryotherapy, radiotherapy, chemotherapy, hormonal therapy; Financing, accessing services for free; Medication: Pharmacies and drug prices; Managing disease; Lifestyle: Nutrition	**Find**	Seek diagnosis, access to health system, care; Seek advice, second opinion; Seek alternative treatment/help from herbalist; Ask information from doctor difficult
**Motivation**	Severity of symptoms as motivators to seek treatment; Supported by others/ receiving information and support	**Understand**	Healthcare structure; Information (inadequately)
**Competence**	In Swahili, digital skills,; Detect signs, symptoms and changes; Seek treatment, accept diagnosis, adhere to treatment, Communication skills: Ask questions and understand answers and results; Make decision, System navigation; Financially: Pay, pay for transport, pay for treatment, pay back; Forge new relationships	**Appraise**	Signs, symptoms and their severity; Judge health care choices
		**Apply**	Sharing information as part of healing process

Although the studies offer a comprehensive overview of pertinent knowledge and health information, they provide minimal insight into the dimensions of active engagement with the information by the population. There is a dearth of information available on how the general public can proactively search and find information on preventive behaviour, nor on how they specifically apply the information found. Some studies report on the ways in which the general public can “understand” messages conveyed by healthcare providers regarding various aspects of the disease, including its severity and relevance of HPV. In other rare cases, the information is appraised in order to make informed decisions regarding the prevention or acceptance of the vaccine.

**Early Detection, including screening**: This category encompassed 52 studies that focused on raising awareness and motivation for screening. Two articles specifically used the terminology of health literacy. In their study on a community health worker-led education programme, Choi et al. (9) employed the term “health literacy” on several occasions and argued that health literacy can be increased through health education, while concurrently addressing beliefs that impede screening uptake. This is particularly pertinent in regions where the provision of health prevention services is constrained. The researchers posited that it is imperative to be cognizant of the pervasive misconceptions and topics that necessitate clarification in order to meet the health literacy needs of the population. In their investigation of factors influencing cancer knowledge in Kenya, Kangmennaang et al. (16) proposed that “access to health literacy through mass media”, a crucial conduit for raising awareness and disseminating information in Kenya, is essential. Several studies indicated that access to health literacy through mass media, particularly television, is often predictive of screening behaviour. Based on Kangmennaang's study, the Kikuyus exhibited the highest levels of health literacy levels in the country.

The remaining studies reported on knowledge, awareness and information about cancer. Furthermore, general information about the health/disease status, risk factors, recommended behaviour/screening, benefits of health interventions (vaccinations, screenings, treatments) and availability and accessibility of services were listed. Some studies also highlighted the relevance of the partners' awareness, for instance in relation to their role in transmitting HPV and post-procedure abstinence (see Table 4 Phase 2).

Furthermore, studies seldom considered the degree to which the general public actively engaged with the information presented. A single study reported on individuals who proactively sought information from the media. The capacity to comprehend information was found to be related to a number of processes, including transmission and reinfection, the relevance of treatment, and outcomes. Some studies included information on the appraisal of the information provided, which related to symptoms, screening behaviour, treatment services and the acceptance of information. The aforementioned studies did not include any commentary on the application or use of the information in question. Nevertheless, some of the behaviours that were requested are reported.

**Treatment:** Five of the 31 studies included in this category explicitly used the terminology of health literacy. Kassaman et al.'s study (22) of the information needs of cervical and breast cancer patients represents the most comprehensive investigation of health literacy among cancer patients in Kenya to date. In her longitudinal qualitative study, Kassaman conducted interviews with breast and cervical cancer survivors, which enabled her to identify and group a number of needs experienced by patients throughout their cancer journey. Henry et al. (84) reported that health literacy, such as lack of terminology, is a patient-related barrier to communicating a cancer diagnosis. Henry et al. established a link between “health literacy” and misconceptions and challenges in reconciling traditional explanatory models of cancer with Western-based medicine. McMahon et al. (62) asserted that health literacy is a pivotal factor in facilitating chemotherapy initiation and adherence. Additionally, they found that health literacy is a crucial element in the socio-ecological model for understanding diagnostic delays (63). Lehman et al. (5) argue that improving health literacy through awareness-raising campaigns that increase knowledge about cancer is a necessity.

The treatment phase [starting from diagnosis (84, 89)] includes the most extensive range of information, knowledge, and skills requirements compared to the previous phases (see Table 4 Phase 3).

While the aforementioned studies seldom elaborate on how the target group should utilise the information, awareness, and knowledge gained, this overview identifies a number of key aspects that are relevant for the processes of finding, understanding, appraising and applying information.

Lastly, the two studies on survivorship indicated that some survivors accessed the internet to obtain information due to their dissatisfaction with the explanation of retinoblastoma. This was driven by a personal desire to understand it better and by a general interest in the subject matter. Furthermore, the studies prompted a discussion encompassing the survivors' knowledge of cancer development, their grasp of the terminology pertaining to genetics and gene and lifestyle factors.

Cancer health literacy during palliative care introduces another dimension, namely the ability to engage with information and knowledge about terminal illness, advance directives and make decisions (66). No details are provided regarding the manner in which the knowledge should be engaged with. The objective is to ascertain the capacity to interact optimally with one's environment.

### 3.3 Situational analysis of cancer health literacy interventions in Kenya

A notable number of studies have examined the factors that impede or facilitate the uptake of specific recommended behaviours, with a particular focus on screening. These studies have identified a plethora of barriers to the recommended health-promoting and help-seeking behaviours. However, for the purpose of this study, data was extracted that was linked to interventions addressing CHL, including the accessing, interpreting, and using of health information. The data was derived from either the 14 implemented interventions or the recommendations provided at the conclusion of the studies (see Table 5).

**Table 5:** Situational analysis of interventions to improve cancer health literacy.

**Factors**	**Sub-themes**	**Prevention**	**Early detection intervention study**	**Treatment intervention study**
**Phase 1: Social assessment**				
Quality of life		Not mentioned	Not mentioned	QoL of patients with advanced cancer from different sociodemographic backgrounds
**Phase 2: Epidemiological, behavioural and environmental assessment**				
Genetics		Not mentioned	Not mentioned	N. m.
Behaviour		Taking child for vaccination, being vaccinated; Limited by competing intentions (other important activities)	Attending session, going for screening, self-examination (physical examination)	Searching information
Environment	Difficulties accessing service	Accessing places of intervention (school)	Proximity to screening sites beneficial	Not mentioned
	Characteristics of care providers	Not specified	Being present, caring, respecting privacy	Not specified
	Internet/TV	Not mentioned	Not mentioned	Widely available internet and extremely good uptake of internet-based resources
Health		Prevent HPV vaccination	Detect cancer early, when its localized	
**Phase 3: Educational and ecological assessment**				
Predisposing factor	Knowledge	Of cervical cancer, HP vaccine, vaccination opportunities when and where; Being well informed	Of cervical cancer, symptoms, risk factors, misconceptions, testing, screening, services offered in local health facility; Causes of breast cancer, cancer presentation signs, high risk groups, screening methods, self-breast exam procedure, treatment options	Psychological needs, needs around daily living and physical support, needs around health care systems; Knowledge about clinical features, pathology, type of cancer, treatment
Enabling factor	Fear/motivation	Acceptance/willingness; Fear of side effects; Susceptibility, self-efficacy	Fear of self-sampling and screening, disease and death; Fear-evoking message proved beneficial; Fear that HPV test interferes with fertility	Not mentioned
	Stigma/misconceptions	Destigmatization needed (e.g. HPV similarly to HIV campaigns); prevents people to go to educational session; stigma due to link to sexually transmitted HPV, association with HIV, fatalistic view of cancer and side effects	Stigma exists: Prevents women from seeking early detection	Not mentioned
	Religious/cultural beliefs	e.g. religious affiliation correlated with HPV uptake, most components of Health belief model,	Not mentioned	Not mentioned
	Money or financial concerns	No financial concerns if intervention in the community	Financial support increases likeliness to undergo screening	Low internet costs
	Trust in health care system & its services	Suggested: trust in health care	Not mentioned	Not mentioned
Reinforcing factor	Support by family	Approval by parent/partner	Spousal approval, partner encouragement	Not mentioned
	Support by social network	Social desirability of vaccination; Support by others (subjective norms)	Social support increases likeliness to undergo screening,	Network of caregivers
**Phase 4: Intervention alignment and administrative and policy assessment**				
Education	Community based	School-based: focus on teachers as gatekeepers to parents and thus children/school vaccination; Community based: community health workers	Community health workers (CHW) administered intervention to increase awareness - facilitate 30-minute interactive talk (on HPV) - intervention in community units (cervical) - intervention during household visits (prostate cancer) - outreach, prior to screening and treatment (breast) Community health campaigns: group education module (e.g. early screening and treatment of breast cancer among women of reproductive age; Cost effective	Educational camps (for metastatic breast cancer patients) + website, interactive forums
	Health care professional	Suggested: support by health providers	Presentation by health professionals (active engagement of participants in small groups, short lecturers by well-trained health professionals, tailored content, presentations and active performing of breast examination; Breast camps at hospital, talks and demonstration of breast examination/or pre-recorded videos or pictorial brochures; Include shared decision-making	Not mentioned
	Online/internet/visual messages	Web-based; Short messages	Short narrated video (loss framed versus gain framed message); Pre-recorded educational videos	Website (see above)
	Others	Health talk	Comprehensive campaign: community, health care professionals, radio, television, social media, advertising material etc.	Not mentioned
Policy		Not mentioned	Screening guidelines	Not mentioned

Phase 1: Social assessment (quality of life and health).

**Quality of life:** although it should be the ultimate goal of all health-related initiatives (see the WHO definition of health (90) it is seldom referenced in studies on the subject. It is only occasionally addressed in studies on treatment (91, 92). It is noteworthy that vaccination and screening were perceived as causing fear and compromising quality of life, rather than enhancing it. Only Caren et al. elucidated the interconnection between information and quality of life, positing that: “Paucity of information was a major challenge, straining relations between caregivers and patients, causing worry to family members and adversely affecting quality of life of the patient (91). This viewpoint was also expressed by Shaikh et al. (82). A multitude of **health**-related factors were identified, contingent on the specific phase of the disease process, including being vaccinated, being detected at an early stage, or health-related improvements during the treatment.

Phase 2: Epidemiological, behavioural and environmental assessment.

**Genetics** was not a focus in any of the intervention studies and it was only addressed in two general studies (26, 65). The review unravelled various **behavioural** aspects that needed to be learned during the different phases including getting vaccinated, breast self-examination, adopting new healthy lifestyles, and coping with the disease. Additionally, these studies highlight the significance of **environmental** factors in three key domains: the accessibility of the intervention, the characteristics of the care provider/intervention facilitator and the digital infrastructure, including the internet or television.

Phase 3: Educational and ecological assessment.

The studies identified numerous **predisposing factors**, including sociodemographic characteristics. However, this study focused on the modifiable factors first such as knowledge and CHL. The various aspects pertaining to CHL were exhaustively discussed in the preceding section.

Overall, **enabling factors** related to five principal domains were documented across all phases and many studies. These factors were linked to a number of emotions such as fear and motivation, beliefs such as social and individual beliefs and stigma, financial aspects and trust in the health system. Emotions were identified as inhibitors, for example anxiety regarding the pain associated with screening or check-ups hindered people to participate in early detection services. However, emotions also served as motivators, as evidenced by the case of individuals who were prompted to attend screening services following the loss of a close friend to cancer. The concept of beliefs and stigma was not only related to HIV, cancer, or skin diseases in general; rather, it could be differentiated along several axes, including self-stigma, perceived stigma, anticipated stigma, and experienced stigma (64, 93). Financial considerations were identified as the primary barrier to accessing screening and healthcare services, with the costs associated with transportation to these facilities and the fees for services. Community-based interventions, for example in schools or local clinics, have reported that financial concerns do not arise in this context. Moreover, several studies have underscored the significance of functional aspects, such as an individual's capacity to attend educational or vaccination sessions or hospital visits. Additionally, the individual's trust in the healthcare system and its services is a pivotal determinant in their willingness to accept the offered services.

Lastly, **reinforcing** factors were linked to social support, specifically from the spouse or a caregiver, parent or guardian. Moreover, the significance of the social context, for instance in reinforcing the uptake of the HPV vaccine or participation in community events for screening, was frequently mentioned. Additionally, social support groups for cancer patients were identified as valuable sources of emotional and instrumental support during the treatment and survivorship phase.

Phase 4: Intervention and alignment and Administrative and Policy Assessment.

**Education**: The most common settings for implementing interventions were the school setting and the community for the prevention phase, the community setting for the screening phase (9, 29, 75), and the healthcare setting for the treatment phase. Other intervention studies employed a range of educational approaches utilising diverse forms of presentation methods, including written material (81), videos or radio/media and websites (82). Furthermore, recommendations presented at the conclusion of other studies underscored the importance of social support groups and individual assistance for cancer patients and their caregivers, along with the potential of mhealth (72).

**Policy**: Despite the existence of numerous **policies**, **guidelines** and **strategies** for cancer treatment and control in Kenya, only a subset of screening interventions make reference to the Kenyan National Cancer Screening guideline (29, 80). A comprehensive list of strategies up until 2018 was provided by Makau-Barasa et al. (30). Most studies make reference to policies and strategies when discussing screening and specific treatments. However, policies regarding education are largely limited to awareness-raising activities.

## 4 Discussion

The objective of this study was to undertake a comprehensive review of the literature conducted on CHL in Kenya, with the aim of synthesising and critically analysing the findings. It should be noted that this review does not encompass the multitude of activities undertaken by individuals and organisations in Kenya with the aim of providing support to cancer patients on a national scale. The majority of these activities are devoid of either regular scientific scrutiny or any scientific basis whatsoever. As a result, this review can only provide an overview of the existing scientific knowledge on CHL in Kenya. To the best of our knowledge, no other review provides an overview of CHL studies in Kenya or any other African country (35). This review provided a comprehensive overview of the methodologies employed in the studies that inform policy-making processes with regard to cancer education and CHL. It shed light on the conceptual framework used and the situational or contextual factors that influence promising interventions. Although the concept of health literacy was already discussed in Nairobi in 2009 at the 9th World Health Promotion Conference (94), it remains a relatively novel phenomenon within the practice, policy and research in Kenya. Accordingly, we elected to adopt a comprehensive scope, consequently also including studies that concentrated on just a single aspect of the holistic concept of CHL.

### 4.1 Limitations

It is important to bear in mind two limitations of this scoping study.

Firstly, it is important to consider the limitations imposed by the **data source**: The data analysed in this study were derived from published research articles and reports in Kenya and not the original raw data. As such, it is not possible to ascertain whether further questions pertaining to CHL or additional associations between factors relevant to cancer health literacy would have been explored. Moreover, as the articles are presented within a specific context for a specific audience employing common vocabulary, it enables the capture of the discourse surrounding them at a particular point in time. The overarching focus on awareness, information, and knowledge is in line with many cancer guidelines but the lack of accessing, understanding, appraising, communicating and applying it without implementing it calls for revisiting semantic understanding and concept conceptualisations and aims of our cancer education interventions. Additionally, as we wanted to describe how CHL is studied and what it contains broadly, we abstained from performing a detailed quality assessment-which is also not a requirement for scoping studies.

Secondly, there is a **paucity of studies** that employ the terminology of cancer (health) literacy and adopt on the **comprehensive approach to the concept** of CHL. This study was exploratory in nature, and thus we included not only studies that used the term “cancer health literacy” in addition as other related terminology, but any study that included many facets related to cancer health literacy, regardless of the terminology used. This broad approach renders comparison complex and challenging, but it does permit the formulation of general observations and the proposal of a CHL model for Kenya. Furthermore, the dearth of existing literature on this topic underscores the necessity for more comprehensive studies on CHL, with a specific emphasis on the ability of individuals to interact with cancer-related information.

### 4.2 Observations about the tradition to research CHL in Kenya

The review examines a specific trend of studies on CHL in Kenya, categorised according to cancer type, phase, and study design. The main focus is on cervical cancer and the prevention and screening of the disease. This is a logical focus, as preventing the disease from occurring and detecting it early are two of the most effective strategies for reducing the overall burden of disease. Surprisingly, prostate cancer, which is the third most prevalent cancer in Kenya and the most prevalent among men, was only the subject of seven studies. This is a relatively limited number, given the relevance of prostate cancer to society. This focus on cervical and breast cancer vs. prostate cancer is not exclusive to Kenya, it is a phenomenon that can be observed across the African continent (95). Additionally, the studies predominantly involve female participants, which is surprising given that studies at all stages of the cancer care continuum indicate that spousal support and approval are crucial factors that warrant further investigation.

The observed inclination to focus on one-point-in-time cross-sectional studies, which represent 90% of all studies, is understandable given the novel status of the phenomenon under investigation. Nevertheless, following over 15 years of research, it has become evident that cancer awareness and knowledge remain low. Consequently, there is a pressing need for more studies that focus on interventions and longitudinal studies. The reintroduction of questions about cancer awareness in the Demographic and Health Survey would facilitate the monitoring of basic cancer awareness over time. However, this review clearly demonstrated that awareness of cancer is insufficient; it is necessary to understand how it is framed. For example, cancer is often presented as a death sentence, and people must be equipped to use the information they receive adequately. This includes the ability to challenge the myths surrounding cancer and to follow the advice provided by experts.

### 4.3 CHL in Kenya

While many studies in Kenya primarily focused on knowledge, some employed a KAP approach, encompassing knowledge, attitudes and practices or even situated information, motivation and behaviour(al skills) (62, 77). Nevertheless, these studies frequently assume, albeit implicitly, that individuals who possess the requisite knowledge and information are inherently capable of utilising them. However, health literacy studies have demonstrated that this assumption is not accurate. Rather, people require the ability, competence, or skills to use the information. It is further recommended that KAP studies are conducted in Kenya (4). However, this study deliberately focused on the abilities of using the presented knowledge in everyday life. The nine studies that employed the term health literacy were all published over the past six years, during which time the concept of HL has been widely embraced globally. It is noteworthy that despite the term “health literacy” being introduced to the global agenda at the 7th World Health Promotion Conference in Nairobi in 2009 (94) and subsequently employed extensively in numerous studies and policies (11, 96), it was only 11 years later that it was utilised in research pertaining to cancer in Kenya (9). The range of studies is diverse and stretches from use of “% ever heard of” as a proxy of “awareness” (77), vs. assessing “knowledge” with more concrete questions about correct/wrong statements. Other studies however use awareness, knowledge or having information interchangeably. Most studies address information, awareness and knowledge provided by others, with minimal attention paid to the process of seeking, appraising and using such information awareness and knowledge. However, Caren stated “information is therapeutic” (91). A person who is cancer health literate is one who has the ability to make informed decisions and choices. The utilisation of Sorensen et al.'s framework (14) to develop a coding scheme for the operationalisation of CHL, the extraction of data and its subsequent analysis proved an effective approach. This approach facilitated the charting and visualising of the existing understanding of CHL in Kenya, with a particular focus on information utilisation. The findings indicated that aspects associated with CHL in Kenya are primarily linked to knowledge, awareness, and information, but not to the ability to utilise the information effectively. In her study of the informational needs of patients with cervical and breast cancer, Kassaman identified numerous themes and grouped them according to the stages of the cancer journey (22, 97). This study represents the most comprehensive examination of breast and cervical CHL in Kenya.

Upon closer examination of the ways in which individuals should interact with information, this review revealed that the studies tend to overlook the process of “finding” or “appraising” information. A greater number of studies concentrated on fostering awareness of behaviours, but fewer investigated whether this was achieved through informed decision-making which is a common phenomenon worldwide, see the CHL scales. Lastly, it is frequently the case that not only the individual in question, but also other family members, etc., are relevant in order to understand and utilise the information in question. Therefore, CHL can be more accurately described as a shared competence. Besides CHL, the “cancer patient activation” debate engages with comparable discussions (76) and refers to the individual's knowledge, skill, and confidence. However, it seems to be constrained to activities associated with clinical care, rather than encompassing the entirety of the cancer care continuum. It is not this author's intention to assert that one approach is inherently superior to all others. Rather, the objective was to identify the most feasible approach for examining how individuals engage with information, specifically in the context of CHL. This study adopted the fundamental tenets of the European Health Literacy framework to delineate the spectrum of CHL activities. This framework is sufficient for the purpose of identifying the information and competences required in general. This framework does not specify the circumstances under which individuals are required to engage with information. This review demonstrated that individuals utilise information in a variety of settings, including at home, in hospital, when interacting with healthcare providers, and when engaging in conversations with others. There is a notable degree of overlap with the Health Literacy Questionnaire proposed by Osborne et al., which has been recommended for use in the management of non-communicable disease (13, 98, 99). The level of health literacy in relation to cancer and other non-communicable diseases is low on a global scale, and similarly low in low- and middle-income countries such as Kenya (100).

**Figure 6:**
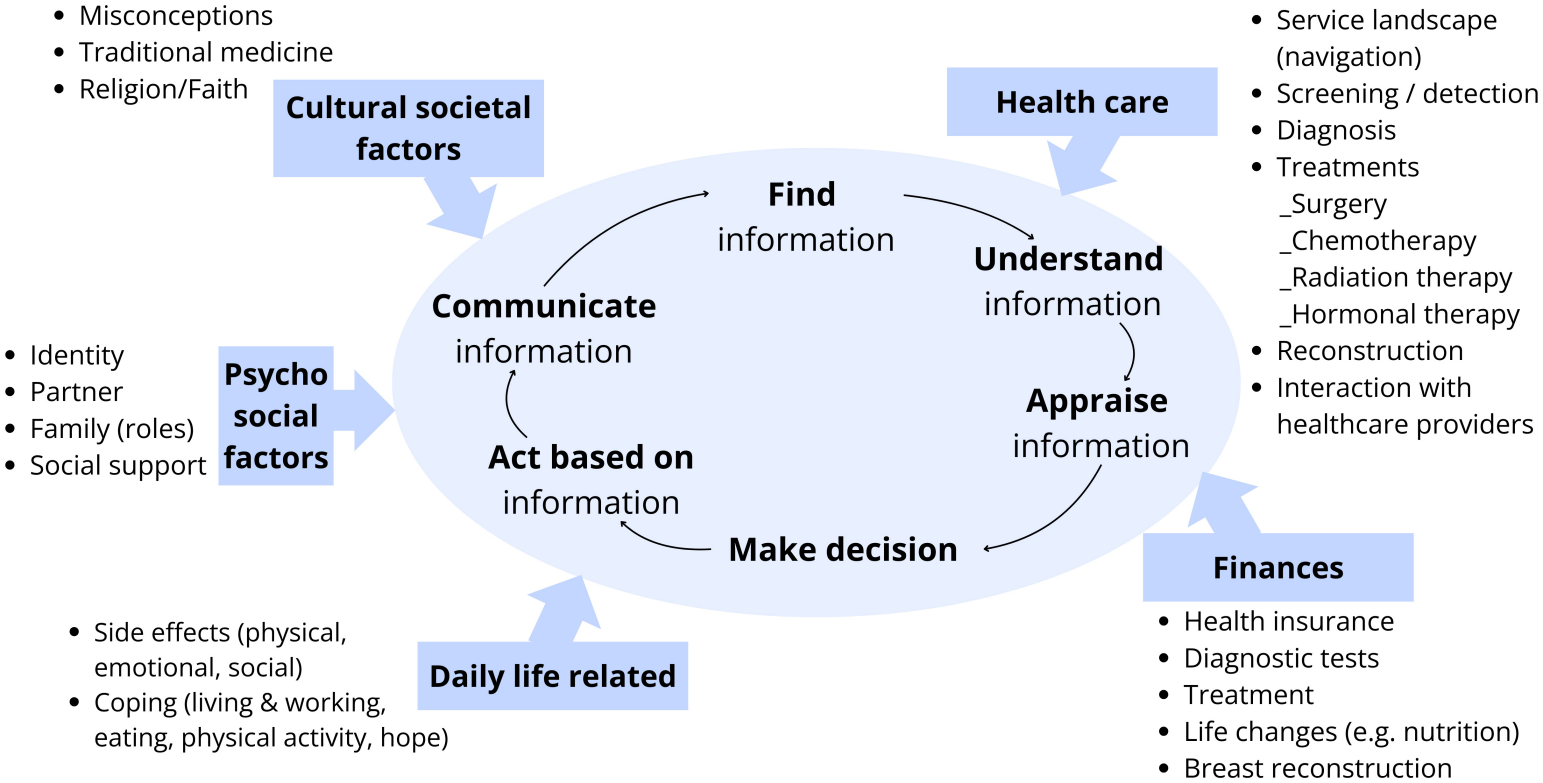
A draft of the concept of cancer health literacy during the treatment phase—based on the research.

Irrespective of the model of health literacy employed, the review identified a research gap pertaining to the manner in which individuals engage with the information they receive and the optimal means of promoting such an engagement within its specific context. The findings of this scoping review, in conjunction with other findings of the CaLioS research project, have the potential to inform the design of a CHL model that is specifically relevant for Kenya. In light of the aforementioned findings, we put forward the following conceptual model of CHL during the treatment phase for consideration (Figure 6). This model will be further delineated in subsequent phases of the research project.

### 4.4 Situational analysis of cancer health literacy improvement in Kenya

While other studies in Kenya have employed the precede–proceed model (88), no study related to cancer in Kenya thus far has done so. The most common methods for exploring environmental or situational factors have focused on barriers and facilitators (28, 84), situated Information, Motivation, Behavioural Skills framework (62) or the socio-ecological model to organise the different factors (22, 64). This review employed the precede–proceed model to identify potentially relevant factors when exploring and promoting cancer health literacy-related factors and to describe the relationship between these factors. This approach can facilitate the development of “culturally grounded communications research and program design“ (101).

It is noteworthy that only studies that concentrate on the treatment and survivorship or palliative phase address the subject of quality of life in relation to health literacy. It is notable that none of the studies focusing on prevention and early detection employ quality of life as the ultimate outcome of their interventions. The assertion, as put forth by the study by Muchiri et al. (73), that interventions based on fear and avoidance rather than positive gains are more successful in Kenya requires further investigation.

It is surprising that there is so little attention paid to *genetics*, given that there are numerous types of cancer that can be inherited and that numerous studies in Kenya have revealed the need for a more comprehensive understanding of genetics and cancer in Kenya. The *behavioural* aspects reveal a variety of aspects, however, there is a paucity of research exploring the influence of lifestyle changes on the delayed onset of non-communicable diseases. The *environmental* aspects related to accessibility of services are not exclusive to a particular region; they are a global phenomenon. Similarly, the importance of the healthcare provider and the recent expansion of internet usage for communication purposes cannot be overlooked.

Health literacy was identified as the main predisposing factor in all its variety and complexity. Several enabling factors were identified that touch upon both emotions but also finances and functionality and can be found in other studies as well. The importance of belief related aspects such as fear/motivation, stigma and misperceptions as well as religious/cultural beliefs and trust in healthcare system combined with finances are important not only in Kenya but also for cancer patients globally. Interestingly, the role of social others, the partner/family as well as the support system played a crucial role in reinforcing the uptake of the ideas. The focus on health literacy as a shared or distributed competence should be further explored (102).

Policies pertaining to cancer control and treatment have been formulated in Kenya, [for details, see the list provided by Makau-Barasa et al. (30)]. However, a comprehensive examination of the educational programmes in question reveals that the prevailing approach is primarily one of information dissemination, rather than one that aims to enhance cancer health literacy. In alignment with cancer education strategies, such as the EU strategy, there is a need to place greater emphasis on CHL and shared decision-making among individuals.

While educational interventions vary in their approach, they encompass a range of strategies, including setting-based approaches, such as those implemented in schools, women's groups, health facilities, or religious gatherings. Additionally, they include public campaigns and the utilisation of community health volunteers (103) or medical professionals, for instance as part of their professional training (104) with the objective of educating patients and the public. Other approaches to disseminating information employ the use of technology as a source of information (105), with television and radio serving as the primary media. In addition, the internet and also social media are becoming increasingly popular as sources of information, particularly among the younger demographics. It is important to consider the barriers to internet usage, as evidenced by a study from 2012 which identified several key factors: inaccessibility of the internet/computer, low or limited computer literacy and operational skills, inadequate infrastructure associated with the same (e.g., lack of electricity) and associated computer costs (105). It is likely that these challenges have been reduced over the past 8 years, for example, with the advent of the smartphone, which is now owned by more than half of Kenyans (106). Gakunga demonstrated in 2023 that television and other media sources were the second most preferred avenue for women and the third most preferred avenue for men to receive information about screening for cancers (107).

REFLECTION on cultural context/cultural features.

Kenya is made of more than 40 tribes and each has its unique *local illness representations* based on the Common-Sense Model of Self-regulation (108), which may influence their response to CHL-related information. A previous study in Kenya suggests that when cancer-related information about some breast cancer symptoms was provided without being sensitive to local illness representations, the information tended to be misinterpreted and local treatment remedies preferred to prompt symptomatic help-seeking (31). While this review demonstrates that CHL-related information in Kenya is either structured (e.g., health campaigns) or unstructured (e.g., social media), there is dearth of evidence on how contextual/cultural factors and individual's characteristics such as self-efficacy beliefs (109) mediated engagement with the information and the resultant cancer-related behaviour. It is noteworthy that several studies incorporated within the present review make reference to culture as a crucial factor in relation to cancer knowledge and CHL. However, a more thorough reading of the concept of “culture” as employed in these studies reveals an absence of precise definitions or concrete examples. Consequently, this review study was unable to provide a comprehensive elaboration on the concept of culture. Further qualitative research is recommended to develop a deeper understanding of the role of culture, including tribal differences, language, and other influencing factors such as religion, educational opportunities, social values, and lifestyle in urban vs. rural settings, in understanding and responding to illness.

The comprehensive analysis of the published studies reveals that cancer health literacy-related aspects are just one aspect, and they require a supportive/fruitful environment to unfold their potential and an increasingly better equipped health care system that can promote health literacy of all its clients.

### 4.5 Recommendations for interventions

It is evident that further studies are required to investigate the determinants of CHL and the impacts (short, medium and long term) of cancer educational interventions. There is a necessity for the adaptation or tailoring of evidence-based educational interventions into the heterogeneous Kenyan context through implementation science efforts based on the influencing factors identified in this review. The cultural diversity of Kenya's multi-ethnic/tribal population must be considered when designing national cancer educational interventions. Drawing from the authors' extensive understanding of various regions in Kenya and the evidence of promising interventions, and inspired by the findings of this review, we recommend that CHL be promoted where people are and through existing groups, communities and health promoters. In this context, religious institutions, including churches and faith-based organisations, can function as pivotal conduits for disseminating information and exemplifying subsequent behaviours related to early screening, acceptance, coping mechanisms, and access to social support. This approach would be in alignment with the values espoused by these institutions, which include stewardship of the body, acceptance of community, and the practice of living in community. The utilisation of these forums would ensure the dissemination of information to individuals of all ages and genders. Moreover, the (bi)weekly meetings of the women's groups, known as “chamas”, could be utilised to enhance CHL. The Kenyan healthcare system's existing grassroots structure, which includes community health volunteers (CHVs) trained to monitor diseases and educate communities, could be utilised to disseminate information on cancer, facilitate understanding, highlight the advantages, and provide concrete methods for implementation. The incorporation of community health promoters presents a notable advantage, given their constant presence on the ground and their capacity to facilitate follow-up discussions with community members, thereby providing further information on cancer. A further avenue that has yet to be thoroughly explored with regard to the promotion of CHL is the establishment of collaborative relationships between cancer support groups and healthcare professionals, including nursing and medical students. Such collaborative efforts could involve the joint organisation of awareness events within communities, for example during the chief “bazaras” (=community gatherings). It is vital to acknowledge that the dissemination of medical information is but one facet of this multifaceted endeavour. The promotion of CHL must encompass a comprehensive approach, encompassing the various domains of CHL, including the financial implications of cancer and strategies for coping with the physical and mental challenges that individuals and families face within their respective socio-cultural contexts.

## 5 Conclusion

The empirical evidence and concepts related to CHL in Kenya are diverse and evolving rapidly. This scoping review offers a comprehensive foundation for an initial overview of research on cancer health literacy in Kenya. The comprehensive analysis permits for the formulation of four recommendations.

Firstly, a considerable corpus of research has already been conducted in this field, although it has primarily focused on cross-sectional studies. This emphasis on cross-sectional studies underscores the necessity for more longitudinal and intervention studies, which can elucidate the temporal evolution of cancer health literacy over time and the efficacy of interventions to enhance it.

Secondly, it is of particular importance to direct attention towards the groups that have been overlooked thus far. It is recommended that particular attention be paid to specific vulnerable or neglected populations, such as those residing in rural areas or living in underserved settings such as slums in Kenya's cities and newly diagnosed cancer patients, with a particular focus on men and caretakers.

Thirdly, conceptualisation of the CHL is a crucial aspect. While knowledge is fundamental, cancer health literacy is essential for the effective translation of the knowledge into practice. This requires a paradigm shift in educational approaches to bridge the gap between theory and practice.

Fourthly, the various situational aspects relevant for interventions on cancer health literacy in Kenya should further be included—both in the interventions but also in the reporting of the interventions—as they might play a crucial role in the actual outcome.

Fifthly, the concept of cancer health literacy in Kenya cannot be considered as an individual phenomenon; rather, it is as a social phenomenon that could be defined as a “social disease”. Cancer has a significant impact on all members of society. The application of knowledge can be conceptualised as a “social practice”, which serves to enhance cancer health literacy (CHL) as a “social engagement”. Furthermore, health literacy can be conceptualised as a shared or distributed competence. It would be advantageous to consider incorporating the social dimension of learning about cancer and developing skills in forthcoming interventions. Additionally, it would be advantageous to leverage the new opportunities afforded by the internet to reach people in rural areas and other underserved settings.

The sharing of lived experiences by cancer patients, survivors or “warriors” as commonly used in Kenya represents a promising approach to addressing the knowledge, motivation and competence required to engage effectively with cancer information (harnessing the opportunities provided by the internet) (33). This facilitates timely access to reliable health information, thereby improving health and quality of life in the long term. The Kenyan guidelines on cancer control constitute a promising foundation for future progress. The integration of health literacy as a core component has the potential to result in an increased number of cancers being prevented, diagnosed at an earlier stage and treated effectively.

## Supplementary material

Supplementary material 1 Search strategy for various databases.

Supplementary material 2 Coding scheme for extracting data.

Supplementary material 3 Data extracted for concept of HL.

Supplementary material 4 Prisma-ScR Protocol.
