# Supplementary material for: Cancer health literacy in Kenya - A scoping review on evidence, concept and a situational analysis of interventions
Source: Front Public Health. 2025 May 16;13:1527400. doi: 10.3389/fpubh.2025.1527400 (PMC12124285; doi:10.3389/fpubh.2025.1527400)
Supplement: Supplementary material 1 — Search strategy for various databases. [file Data_Sheet_1.docx]

Supplementary Material 1: Search Strategy to identify studies for potential inclusion

# Web of Science (last searched on 2024/02/05)

Search string: AB=(cancer or oncology or HPV ) AND AB=(Kenya Or Kenyan* ) AND AB=(knowledge OR understanding OR awareness OR belief* OR perception* OR behaviour OR behavior OR practice* OR experience* OR skill* OR competenc* OR literacy OR competencies OR capabili* OR abilit* OR coping OR motivation )

Limitations: Range 2010-01-01 to 2023-10-30

# Pubmed (last searched on 2024/02/05)

((Kenya[Title/Abstract] OR Kenyan*[Title/Abstract]) AND (cancer[Title/Abstract] OR oncology[Title/Abstract] OR HPV[Title/Abstract])) AND (knowledge[Title/Abstract] OR understanding[Title/Abstract] OR awareness[Title/Abstract] OR belief*[Title/Abstract] OR perception*[Title/Abstract] OR behaviour[Title/Abstract] OR behavior[Title/Abstract] OR practice*[Title/Abstract] OR experience*[Title/Abstract] OR skill*[Title/Abstract] OR competenc*[Title/Abstract] OR literacy[Title/Abstract] OR competencies[Title/Abstract] OR capabili*[Title/Abstract] OR abilit*[Title/Abstract] OR coping[Title/Abstract] OR motivation[Title/Abstract])

Filters: from 2010/1/1 - 2023/10/30

# Cinahl, PsycInfo, Eric, OpenDissertation using EBSCO host (last searched on 2024/02/05)

AB ( Kenya Or Kenyan* ) AND AB ( cancer or oncology or HPV ) AND AB ( knowledge OR understanding OR awareness OR belief* OR perception* OR behaviour OR behavior OR practice* OR experience* OR skill* OR competenc* OR literacy OR competencies OR capabili* OR abilit* OR coping OR motivation )

Restricted to: 2010-2023

# African Index Medicus

(tw:(knowledge OR understanding OR awareness OR belief* OR perception* OR behaviour OR behavior OR practice* OR experience* OR skill* OR competenc* OR literacy OR competencies OR capabili* OR abilit* OR coping OR motivation)) AND (tw:(cancer or oncology or HPV)) AND (tw:(Kenya or Kenya*))

Year Range 2010-2023
