# Supplementary material for: Cancer health literacy in Kenya - A scoping review on evidence, concept and a situational analysis of interventions
Source: Front Public Health. 2025 May 16;13:1527400. doi: 10.3389/fpubh.2025.1527400 (PMC12124285; doi:10.3389/fpubh.2025.1527400)
Supplement: Supplementary material 2 — Coding scheme for extracting data. [file Data_Sheet_2.docx]

Annex 2: coding schemes & MAXQDA coding scheme

# Coding scheme 2A: Cancer health literacy

| **Cancer Literacy Domain** | **Term** | **Concretely** |
| --- | --- | --- |
| Knowledge/awareness | knowledge | This code contains any evidence that refers to the knowledge that the public or cancer patients need – specified by ‘knowledge’ related to . |
|  | awareness | This code contains any evidence that refers to the awareness that the public or cancer patients need – specified by ‘awareness’ related to or on. |
|  | information | This code contains any evidence that refers to the information that the public or cancer patients need – specified by ‘information related to |
| Motivation | (e.g. beliefs, susceptibility | This code contains any evidence that refers to the motivation, beliefs or susceptibility that the public or cancer patients need – specified by ‘motivation’ related to/on . |
| Competence | competence | This code contains any evidence that refers to the competences and its synonyms that the public or cancer patients need – specified by ‘competences’ related to/on. |
|  | ability | This code contains any evidence that refers to the ability and its synonyms that the public or cancer patients need – specified by ‘ability’ to |
|  | skills | This code contains any evidence that refers to the skills and its synonyms that the public or cancer patients need – specified by ‘skills to |
| -find | Find, access, seek | This code contains any evidence that refers to the competence of finding information and related verbs (e.g. access/ seek) that the public or cancer patients need – specified by ‘find information’ |
| -understand | Understand, comprehend | This code contains any evidence that refers to the competence of understanding information and related verbs (e.g. comprehend) that the public or cancer patients need – specified by ‘understand information’ |
| -appraise | Appraise, accept, judge | This code contains any evidence that refers to the competence of appraising information and related verbs (e.g. appraise/accept) that the public or cancer patients need – specified by ‘appraise information’ |
| -apply | Apply, use | This code contains any evidence that refers to the competence of applying information and related verbs (e.g. use) that the public or cancer patients need – specified by ‘apply information’ |

# Coding scheme 2A: Contextual analysis of interventions to promote cancer health literacy

| **Constructs** | **Possible Factors** | **Key findings** |
| --- | --- | --- |
| ADMINISTRATIVE AND POLICY ASSESSMENT | | |
| Policy, regulation, organisation | any policy strategy  existing  recommended | List any information that reports on policies, regulations or strategies that aim at improving cancer literacy (or a subcomponent of it) |
| Health education | types of education  performed  recommended | List any information on interventions that aim at improving cancer health literacy (or a subcomponent of it) |
| EDUCATIONAL AND ECOLOGICAL ASSESSMENT | | |
| Predisposing Factors | knowledge, attitudes, beliefs, cultural values, perceptions, where he also refers to as the motivational aspects (information, cognitive processing, rational, emotional, motivational) | List any information that is linked to cancer health literacy (and its subcomponents) |
| Enabling Factors | availability of resources, accessibility | List any information that is linked to enabling factors such as fear/motivation, stigma / misconceptions, religious or cultural beliefs, financial factors, trust |
| Reinforcing factors | influence from parents, teachers, employers, peers to be awarded/praised/judged | List any information linked to reinforcing factors e.g. support by the family or support by the social network, or also messages from TV or radio |
| BEHAVIORAL AND ENVIRONMENTAL ASSESSMENT | | |
| Genetics | hereditary cancer | List any information on genetics that might be relevant for the intervention |
| Behaviour | All behaviour that cancer patients need to exhibit from attending prevention campaigns, getting vaccinated, getting screened, attending treatment | List any behaviour that is reported which is addressed or relevant for the intervention |
| Environment | Availability of cancer centres and services, transport and internet | List any information regarding the environment that becomes relevant for the intervention |
| EPIDEMIOLOGICAL ASSESSMENT | | |
| Health | Health / disease | List any information regarding health that was targeted in the intervention |
| SOCIAL ASSESSMENT | | |
| Quality of life | Quality of life | List any information regarding quality of life and how the intervention improves it. |

# MAXQDA coding scheme


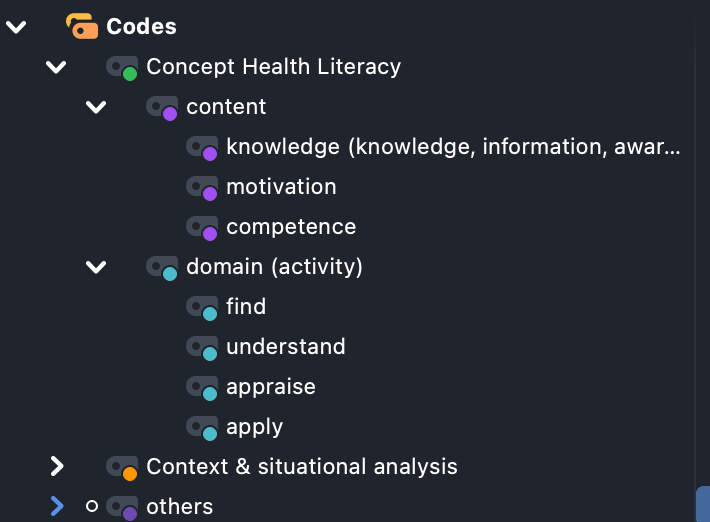


**Supplementary Figure 2a.** Coding scheme in MAXQDA for the concept of health literacy


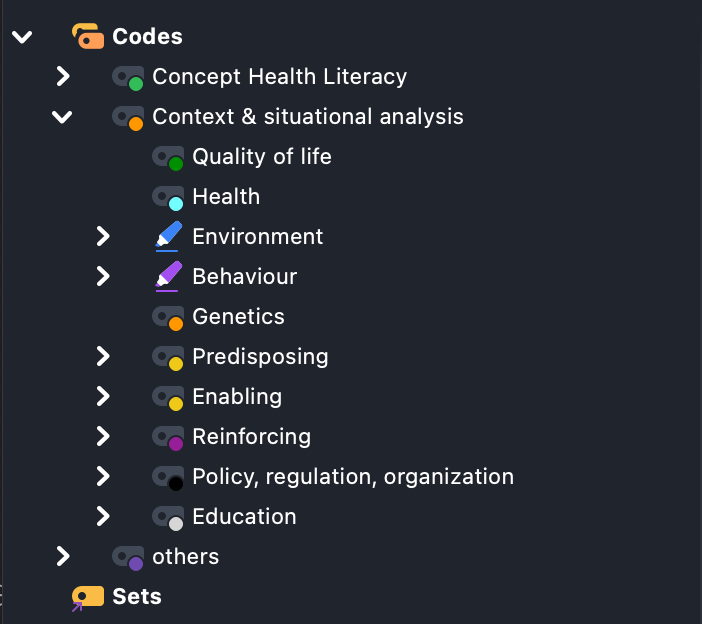


**Supplementary Figure 2b.** Coding scheme in MAXQDA for the PRECEDE-model
