# Supplementary material for: Cancer health literacy in Kenya - A scoping review on evidence, concept and a situational analysis of interventions
Source: Front Public Health. 2025 May 16;13:1527400. doi: 10.3389/fpubh.2025.1527400 (PMC12124285; doi:10.3389/fpubh.2025.1527400)
Supplement: Supplementary material 3 — Data extracted for concept of HL. [file Data_Sheet_3.docx]

Supplementary File 3 components of the concept of cancer health literacy

# Prevention

| **Cancer Literacy Domain** | **Term & number** | **Findings themes** | **Findings** |
| --- | --- | --- | --- |
| Knowledge/awareness | knowledge | **Human body:** cervix, the normal appearance of a breast, biological knowledge  **Cancer and specific types** of cancer, e.g. cervical cancer, oesophageal cancer, breast cancer  **Symptoms of cancer & side-effects**  **Cause of cancer:** Human Papilloma Virus, transmission, link between HPV and cervical cancer  **Risk factors**: early sexual debut, smoking, having multiple sexual partners, minimal symptomatic in an early stage, HPV infection, geography  **Prevention modalities:** Vaccination, pap smear test (goal)  **Services:** regulation: HPV vaccination for target group (girls aged 10 and above), service for free, where and when available  **Treatment modalities:** chemotherapy, radiation, and surgery  **Characteristic of the information:** Accurate / right / credible / proper  Misinformation: e.g. HPV is a form of contraception encouraging pre-marital sex (Kolek)/ rumour around infertility | **Cancer and specific types** of cancer, e.g. cervical cancer, oesophageal cancer, breast cancer  late health-seeking behaviour  Cervical cancer facilities  HPV vaccine  HPV as a sexually transmitted virus by unvaccinated people  Goal of HPV vaccination (to prevent cervical cancer)  Pap smear & goal (to prevent invasive cervical cancer)  Prevention of cervical cancer and HPV  Biological knowledge  Cervix  Cause of cervical cancer  How normal breast looks like  **Risk factors**: early sexual debut, smoking, having multiple sexual partners, minimal symptomatic in an early stage, HPV infection  **Symptoms/sign** of cervical cancer  Vaccination options: when and where  Screening options (and availabilities)  HPV vaccine protects against Cervical Cancer, Vulvar Cancer, Anal Cancer, Warts, HPV infects both men & women,  **HPV transmission**: through sexual contact, physical contact  **Treatment modalities:** chemotherapy, radiation, and surgery  Consequences of cervical cancer (expressed a desire)  Impact of cervical cancer on community (expressed a desire)  Alternative prevention strategies (expressed a desire)  Oesophageal cancer is common in the area (Bomet), a leading cause of cancer death  Where to get condoms |
|  | awareness |  | Cancer  Cervical cancer  HPV  HPV vaccine  Cervical cancer screening  HPV linked to cervical cancer  HPV is a sexually transmitted disease  Vaccination opportunity  HPV vaccine offered to all girls 10 and above  Services: The government’s launch of the HPV vaccination campaign (targeting all grade four girls)  **Service and modality**: Screening (oesophageal cancer) is free  Severity & poor prognosis (oesophageal cancer)  Lack of a. of program  Misconceptions about HPV/damaging rumours |
|  | information |  | Cervical cancer  Lack of information for decision making  Where and when to get the vaccine  About program  Role of screening and HPV  Preventive breast cancer measures  Breast cancer detection |
| Motivation | (e.g. beliefs, susceptibility | Seriousness of the threat to their health  Willingness to vaccinate  Health belief model  Beliefs about HPV  Vaccine hesitancy  Acceptability of vaccine  To exhibit non-risky behavior  Friends as source of motivation  For online health information seeking  Accurate / right / credible / proper information  perceived risk, seriousness, self-efficacy, response efficacy and response cost (Muturi - 2020 - eHealth literacy and the motivators for HPV preven, S. 3) | Seriousness of the threat to their health  Willingness to vaccinate  Health belief model  Beliefs about HPV  Vaccine hesitancy  Acceptability of vaccine  To exhibit non-risky behavior  Friends as source of motivation  For online health information seeking  Accurate / right / credible / proper information |
| Competence | Competence/ ability / skills | Self-efficacy  **Functional:** to read/ to seek health information online (Muturi 2020)  **decision-related:** to make decision about breast health/ to refuse unsafe sex with husband or partner  **evaluate**: To appraise threats / coping, to evaluate and  use: online health information  **accessibility**: to access condoms  **communication** skills (of health care providers) | Only linked to self-efficacy: ‘expectancies about one’s own competence to perform the behavior’ Vermandere 2016  To appraise threats (Muturi 2020)  To appraise coping (Muturi 2020) |
|  | ability |  | Self-efficacy  Functional: to read/ to seek health information online (Muturi 2020)  decision-related: to make decision about breast health/ to refuse unsafe sex with husband or partner  To appraise threats / coping  accessibility: to access condoms |
|  | Skills |  | Communication skills (specifically health professionals)  Literacy & computer operational skills  skills to evaluate and effectively use online health information in HPV prevention (Muturi - 2020, S. 12) |
| _find | Find, access, seek | Information,  medical attention/ treatment,  permission,  online-health seeking | Not found  Seek right information Alago#  Seek medical attention/treatment (Busolo#)  Online health seeking behavior  Seeking permission  Seek pap screening |
| _understand |  | General: public health information  The disease  Severity/gravity of cancer,  the importance of HPV vaccination | Gravity of cancer and the poor prognosis (oesophageal cancer)  Severity of cervical cancer to health  the disease  the importance of HPV vaccination  Public health messages |
| _appraise | Appraise, accept | Make informed decisions, regarding preventive breast cancer measures  Acceptance of HPV vaccine  Treats & copings  Evaluate online health information | Make informed decisions regarding preventive breast cancer measures  Acceptance of HPV vaccine,  Threats & copings (Muturi et al) |
| _apply | Apply, utilize, use i/k/a | Use online health information | Not found |

# Early Detection

| **Cancer Literacy Domain** | **Term** | **Themes** | **Findings** |
| --- | --- | --- | --- |
| Knowledge/awareness | knowledge | (health concern related or process related)  Health status/disease status  HPV/Cervical cancer (symptoms  Prevention  Screening (procedures  HPV development | Screening methods  Health status  cancer  HPV (progression from HPV to cervical cancer, HPV testing, vaccination)  Cervical cancer (definition, progression, symptoms, treatment, statistics)  CC-related factors  HPV-related factors  Location for CC screening  Breast cancer (source of information)  Prevention (BC)  Risk factors (BC): misconceptions and risk factors  Risk factors (PC  Suggested behaviour (PC/CC)  Screening (BC)/screening methods  Importance of early breast cancer screening /Benefits  Cervical cancer prevention  Impact of cervical cancer  How to seek treatment  Prostate cancer   - Screening (Kassaman 2022) - BSE (Kassaman 2022) - CBE (Kassaman 2022) - Risk factors of BC (Kassaman 2022) - signs and Symptoms of breast cancer (Kassaman 2022) and how the breast normally looks and feels - Diagnostic tests (Kassaman 2022) - Financial implications (Kassaman 2022) - Knowledge of Pap Smear tests (Mabeya et al. - 2018)   HPV knowledge among caregivers (Mabeya et al. - 2018) |
|  | awareness |  | Partner awareness on availability of treatment  HPV  Cervical cancer  CC related factors  Prostate cancer  Partners’ awareness on their role in transmitting HPV  Self-sampling  Own HPV status  Where to access screening/ Of breast screening events  Treatment available  Treatment attendance  Aware of health status (e.g. HPV, own breast)  Cervical cancer prevention  Cervical cancer screening  Risk factors  Pap smear  Visual inspection with acetic acid (VIA)  Prostate cancer screening  Dispelling myths  Of self & perceived choice  Awareness of one wife’s suffering will motivate husbands to support his wife financially |
|  | information |  | Benefits of early detection of cervical cancer  cancer  HPV  HPV and fertility  Mode of transmission,  Association between HPV and cervical cancer  Cervical cancer  Cause of cervical cancer  HPV testing (self-collected: step-by-step description, testing kit, benefit of early detection)  Screening (precancerous screening: Pap smear, VIA/VILI, HPV testing, timing of screening, notification of results)  Signs and symptoms of positive HPV, source of infection,  Screening (availability, where in the hospital, treatment cost, date/when, what to expect, procedures/duration and benefits)  Treatment (recommended HPV treatment)  Follow-up procedure/post-screening (follow-up care  Partner: post-procedure abstinence  Anatomy of the female reproductive system  Target group-specific information (age and social economic status)  Preventive services  Importance of maintaining good health (diet, lifestyle, stress management, physical fitness, regular health checks, breast cancer checks)  Campaigns  Link between screening and early detection  Health issues affecting the community |
| Motivation | (e.g. beliefs, susceptibility | To initiate preventive behaviour/to go for screening,  Fear of cancer hampers/ motivates going for screening | Of young adults to initiate preventive behaviours  Attitude towards CC-related factors  Fear of possible cancer motivates  Motivation linked to Social identity included feelings of privacy and comfort conducting the HPV self-sampling  For screening  Lack of motivation |
| Competence | competence | Perform self-examination,  Accept and complete HPV self-sampling  Access information, seek services, respond to questions | Confidence to detect and report any abnormalities (breast)  no |
|  | ability |  | To convey post-treatment recommendations  To access multiple testing and treatments as the primary reason  To access information from the media  To seek cervical cancer services  To respond to the question about cervical cancer screening/about signs and symptoms  To complete HPV self-sampling strategy  To read, write, send, and receive SMS text messages (for mHealth) |
|  | skills |  | Acceptability of HPV self-sampling,  Confidence in their ability to complete HPV self-sampling strategy  Perform BSE and CBE  Nontechnical skills like communication (health professionals) |
| -finding/accessing |  | Seek & access information (also through media, radio)  **Seek permission** to attend services  Seek information by partner (not done) | Access information from the media (Muthoni and Miller 2010)  Seeking permission to attend services  Seek help (alone)/Pap screening  Seeking information about HPV screening/treatment services (not done by male partners) |
| -understand |  | HPV transmission,  Importance of treatment  Possibility of re-infection  Results | HPV transmission  importance of treatment  re-infection possibility  results |
| -appraise/accept |  | Symptoms  Preventive behaviour & accept screening  Accept educational message  Accept health service, screening & screening methods  Different information | Symptoms (Kailemia et al.#)  Accept preventive behaviour  Willingness to accept screening  Accept health services / accept educational messages  Accept screening method |
| -apply |  |  | N/A |

# Treatment

| **Cancer Literacy Domain** | **Term** | **Themes** | **Findings** |
| --- | --- | --- | --- |
| Knowledge/awareness | knowledge | Nature of disease: signs, symptoms, progression  Benefits of screening, early detection  Accessing treatment: navigating hospital, Alternative places to receive treatment  Results  Treatment modalities: cryotherapy, radiotherapy, chemotherapy, hormonal therapy  Financing, accessing services for free  Medication: Pharmacies & drug prices  Managing disease  Lifestyle: Nutrition | Cancer origin, risk factors, preventive factors,  Various types of cancer, incl. colon cancer  Some are hereditary illness  Screening, tests, investigations  breast cancer pathology/clinical features, types, progression  treatment they were receiving, and different treatment modalities: chemotherapy, radiotherapy, surgery  side effect management  finances  diet, medical powers of traditional vegetables  little knowledge about cancer support groups, free activities, benefits provided by cancer support organisations  right to ask questions  not knowing another patient/person who had cancer before |
|  | awareness |  | Cancer (in general)  Symptoms  Severity  Cancer as non-communicable disease  Benefits of early detection  Screening  Stage of cancer  Support groups (source of information, practical tips)  Treatment: long, complex, costly  Importance to take care of themselves  Relevance of eating healthily  Coping mechanism  Services  How to seek information |
|  | information |  | True nature of disease / causes  Health and personal records  Breast cancer  screening  Cervical cancer  Cervical cancer screening & cryotherapy  Cancer and physical suffering  Importance  Accessing services for free  Financing, low-cost accommodation  Treatment & treatment options: radiotherapy, chemotherapy, side-effects, duration of treatment  Hospital navigation,  Nutrition (also in rural area)  Pharmacies & drug prices  Management  Implications of their illness, also its effect on mobility, ability to work or carry out household tasks and other meaningful activities; ways to improve quality of life  Practical information: health facilities, support groups  Information about existential questions ‘why’ and ‘why me’ |
| Motivation | (e.g. beliefs, susceptibility | Severity of symptoms as motivators to seek treatment  Supported by others/ receiving information and support | - improved by social others - severity of symptoms as motivator to treatment |
| Competence | competence | In Swahili, digital skills,  Detect signs, symptoms and changes  Seek treatment, accept diagnosis, adhere to treatment,  Communication skills: Ask questions & understand answers & results  Make decision,  System navigation  Financially: Pay, pay for transport, pay for treatment, pay back  Forge new relationships | In computer skills/Kiswahili  To make decision for themselves |
|  | ability |  | To fight for life  Detect changes in breast  Cite HPV as a cause of cervical cancer  Seek treatment  Coping  Accept diagnosis  Review their results  Follow up with treatment  Overcome barriers to treatment  Ask & have their questions answered  & capacity to use information  Financially: Pay, pay for transport, pay for treatment, pay back  Behavioural skills/normal daily activities  To eat well (impeded by mouth soreness and blisters)  Initiate and adhere to chemotherapy  Navigate the logistics  Impacted: to eat, concentrate, sleep, interact with others  Forge new relationships  Lost ability to contribute to informal social security through reciprocity  Lost ability to have children |
|  | skills |  | **System navigation skills**: patient’s ability to navigate the logistics of chemotherapy and the complex referral system (McMahon et al. - 2022 - Barriers and facilitators to chemothera, S. 13)  Communication and empathy skills (required from health care providers), family centred communication skills  Computer skills  **activation skills** which facilitated chemotherapy were coping skills, resilience, garnering social support, prioritizing oncology care, acceptance of disease, and  accepting some of the hassles of the healthcare system such as long lines and wait times from treatment (McMahon et al. - 2022 S. 12)  detect cancer  skills to exhibit behavior to response to noticing breast changes  **Functional skills** which facilitated chemotherapy were setting and remembering chemotherapy appointment times, arranging leave for work for chemotherapy, arranging money for transportation and chemotherapy costs, and communicating concerns and wishes to their healthcare provider (McMahon et al. - 2022 - S. 12)  Stress management techniques |
| -find/access | Seek  Find  access | Seek diagnosis, access to health system, care  Seek advice, second opinion  Seek alternative treatment/help from herbalist  Ask information from doctor difficult | Seek second opinion  Seek access to health systems |
| -understand |  | Healthcare structure  Information (inadequately) |  |
| -appraise/accept |  | Signs, symptoms & their severity  Judge health care choices | Breast cancer symptoms/  Severity of their breast cancer symptoms  Judge health care choices |
| -apply |  | Sharing information as part of healing process | Not found  ‘sharing information as part of the healing process’ |
